# Antiretroviral Therapy Adherence Interventions in the Era of Universal Test and Treat: A Hybrid Systematic-Narrative Literature Review of Global Evidence

**DOI:** 10.1007/s10461-025-04867-9

**Published:** 2025-10-06

**Authors:** Claire M. Keene, Lauren Jennings, Carl-Oscar Källström-Ståhlgren, Ingrid T. Katz, Lora L. Sabin, Chantel Schreuder, Yashna Singh, Catherine Orrell, Rivet Amico

**Affiliations:** 1 Nuffield Department of Medicine, Centre for Global Health Research, University of Oxford, Oxford, UK; 2 Desmond Tutu HIV Centre, Institute of Infectious Disease and Molecular Medicine & Department of Medicine, University of Cape Town, Cape Town, South Africa; 3 Department of Public Health, Global Health Section, University of Copenhagen, Copenhagen, Denmark; 4 Brigham and Women’s Hospital, Harvard Medical School, Boston, USA; 5 Department of Global Health, School of Public Health, Boston University, Boston, USA; 6 South African Medical Research Council, Cape Town, South Africa; 7 Department of Health Behavior Health Education, School of Public Health, University of Michigan, Ann Arbor, USA

**Keywords:** HIV, Antiretroviral therapy, Adherence, Intervention, Viral suppression, Universal test-and-treat, Literature review

## Abstract

An undetectable viral load (VL) in people living with HIV (PWH) is key to both individual and public health success. But for the millions of PWH on oral antiretroviral therapy (ART) worldwide, this requires consistent, sustained adherence. Review of interventions to support adherence published in recent literature can provide insights into promising and effective strategies. We conducted a hybrid systematic-narrative literature review to explore optimal adherence strategies in the era of universal test-and-treat. We searched PubMed, Scopus, and Web of Science according to PRISMA guidelines for peer-reviewed studies, available in English, including people ≥ 12 years old taking ART, published between 01 January 2015 and 18 January 2024. We extracted data on the included studies and the adherence interventions (strategies used —allocated to one of 14 a priori categories or ‘other’, measures of adherence, and intervention outcomes). Descriptive statistics were used for study information and those interventions with a positive effect were described narratively. We extracted data from 230 studies evaluating a total of 262 interventions among 97,037 PWH. Most studies enrolled participants in Africa (106, 46%) or North America (80, 35%). The majority randomized participants (215, 94%), including 30 cluster-randomized trials. Most included general HIV clinic populations, with 51 (22%) focused on youth and 23 (10%) on pregnant and post-partum women. Many (146, 64%) used VL as an outcome. Self-reported adherence was also a commonly used outcome (129, 56%), but a minority used self-reported measures alone (36, 16%). The most common intervention strategies included across the 262 interventions were eHealth/ mHealth technologies (90, 34%) and adherence-focused counseling (81, 31%). The majority of interventions had ‘other’ intervention features (133, 51%), typically combined with one or more of the a priori-defined strategies (107, 80%). Most studies evaluated an approach with multiple strategies packaged into a single intervention (k = 182/262, 70%).The majority of interventions had some evidence of effect on an adherence outcome (k = 159, 61%). In studies reporting VL outcomes, 52% (k = 87/166) found some evidence of effect, while 28% (k = 47/166) found significant effects. Intervention strategies demonstrating significant impact on VL included task-shifting and changing dispensing schedules (3/5, 60% in both), while nearly half the evaluations of economic strategies demonstrated significant impact on VL (10/21). A number of different adherence intervention strategies have the potential to impact viral suppression in different populations. Variability in intervention strategies and the resulting outcomes, supports calls to target interventions to PWH who are most likely to benefit, while at the same time addressing social determinants of health and reducing barriers to accessing care to make services more person-centered. Greater attention to evaluating flexible, tailored, complex interventions may offer valuable insights for moving towards the next generation of highly generalizable, sustainable adherence support.

**Supplementary Information:**

The online version contains supplementary material available at 10.1007/s10461-025-04867-9.

## Introduction

Antiretroviral therapy (ART) has dramatically altered clinical and psychosocial outcomes for people with Human Immunodeficiency Virus (PWH). Although there remains no cure for HIV, effective ART has changed this almost uniformly fatal transmissible disease into a manageable chronic condition with a near-normal anticipated lifespan [1], and near-zero odds for onward transmission when the virus is controlled [2, 3]. Viral suppression is thus key to individual and public health benefits. However, for PWH on oral ART regimens this requires consistent adherence to ART to sustain treatment success [4]. This makes effective interventions to improve ART adherence a crucial component of the HIV response.

Positive health outcomes are contingent on adherence: i.e. following prescribed ART instructions. Despite the recent development of long-acting injectable formulations for HIV treatment, the vast majority of the 39 million PWH need to take oral therapy every day [5]. Even with simplifications in ART regimens and widescale availability of more tolerable, once-daily treatment that is increasingly robust to resistance, adherence remains challenging for a variety of reasons [6–8]. 

The global push to end the AIDS epidemic as a threat by 2030, supported by the World Health Organization’s 2015 guidelines on universal test-and-treat and subsequent global shift in treatment policy, relies on achieving the UNAIDS’ 95-95-95 targets. However, globally only 71% of PWH are virally suppressed [9]. As funding for HIV becomes more limited [10], it is increasingly important to utilize state of the science on adherence-support interventions to gain insights on the most effective approaches to assist PWH to reach and sustain viral suppression.

Previous literature analyzing adherence interventions has highlighted a range of promising approaches, including comprehensive complex interventions prioritizing the whole person. However, this literature was dominated by self-reported adherence outcomes, which can be unreliable [11–13]. Over the past decade, viral load (VL) measures have become more feasible to collect and use as the primary outcome of interest across interventional research studies. As a result, there is now a robust evidence base for adherence-related interventions that seek to promote viral suppression for those on daily ART regimens.

This hybrid systematic-narrative review of the ART adherence interventions published since 2015 summarizes the state of the literature, with emphasis on adherence interventions and strategies that demonstrate promise on VL outcomes for PWH on daily oral ART in the era of universal test-and-treat.

## Methods

### Study Design

A hybrid systematic-narrative review [14] was conducted to map, summarize, and categorize interventions to improve adherence to ART in the era of universal test-and-treat, when all PWH are eligible for ART regardless of disease stage or CD4 count. A systematic-narrative hybrid review adopts the rigorous methodology of systematic reviews in the literature identification process, application of inclusion/exclusion criteria, and data extraction procedures. It also utilizes the broad summary approach to synthesize the depth of observations used in narrative reviews. The research team included all authors, comprised of colleagues with extensive experience in the field of HIV adherence, and who adopted a rapid review approach. To enhance rigor and replicability, particularly given the large team, we developed a search strategy, conducted the search and extraction, and reported the review in accordance with Preferred Reporting Items for Systematic Reviews and Meta-Analyses (PRISMA) guidelines [15]. The identified literature was summarized as a narrative to describe the scope of interventions and measures of adherence in order to draw insights and guidance for future directions, without evaluating the quality and strength of evidence [16, 17]. 

### Search Strategy and Selection of the Evidence

A wide search strategy was developed using key phrases to identify literature describing interventions to improve or sustain ART adherence among PWH. The study used the search parameters and limits set out in Table 1 to search PubMed, Scopus, and Web of Science on 18 January 2024. The full search strategy for each database can be found in Supplementary material File A.

**Table 1:** Summary of search parameters and limits as well as final inclusion and exclusion criteria, categorized according to the ‘PICO’ search framework

	Inclusion	Exclusion
Search parameters and limits		
	Published in English	Published in languages other than English
	Published between 1 January 2015 and the date of the search (18 January 2024)	Published before 2015
Eligibility		
Population	Individuals on lifelong ART or who have initiated ART previously (includes PMTCT option B+)	Pre-ART initiation or people on pre- or post-exposure prophylaxis
	Adults and adolescents ≥12 years old	Children < 12 years (unless included in a broader study population that also encompassed adolescents ≥12 years
Intervention	Any intervention targeted at improving adherence to ART, whether directly or indirectly	Intervention not targeted at improving adherence
Control	Had a comparison condition, whether a control arm or a pre-post study	No comparison condition
Outcome	Adherence measured by direct measures of adherence (e.g. using electronic monitoring, pill refill, self-report) or VL / CD4 cell count as a proxy	Adherence or VL not reported, e.g. only measured retention, feasibility, or acceptability
Study characteristics	Peer-reviewed publications presenting intervention(s) outcome(s)	Letters, commentaries, editorials, opinion pieces, conference abstracts, case reports, systematic reviews and protocols. Retrospective observational studies with no control/comparison or secondary analyses

*ART* antiretroviral therapy, *PMTCT* prevention of mother to child transmission, *VL* viral load

Following the search, all identified citations were collated and duplications removed using Covidence software, which was then used to screen the evidence for inclusion. Covidence is a web-based collaboration software platform that streamlines the production of systematic and other literature reviews [18]. The titles and abstracts, and then the full texts, were each screened by two author members of a team of nine reviewers using the criteria outlined in Table 1. The team held weekly meetings to discuss the application of the eligibility criteria. Disagreements were resolved through discussion and consensus, and adjudicated by a third reviewer. Reasons for exclusion were noted at the full-text screening stage.

### Data Extraction, Charting and Synthesis

The data from published articles was extracted using a template in Covidence, with one extractor per included text. Coders met weekly to discuss the extraction template and decision rules. Studies were characterized in terms of publication year, location (continent and country), study design, power and sample size, populations of focus, and inclusion criteria. Intervention strategies, features, and components represented in the literature were summarized overall and in relation to outcomes. The data extraction template and list of a priori categories of intervention strategies can be found in Supplementary material File B.

**Active Adherence Interventions** in each study were characterized (including the main intervention components and specific adherence strategies) using an a priori coding approach based on previous reviews and the initial screening of titles and abstracts in this review. Interventions were coded for inclusion of the following: economic approaches to incentivize adherence or address economic instability, eHealth/ mHealth strategies, treatment or counseling for drug or alcohol use, use of adherence clubs, adherence related education/information provision, adherence counseling approaches adapted or created specifically to support individual-level drivers of adherence, mental health counseling or strategies focused on improvement in well-being, use of peers (as interventionists or delivery in groups), electronic dose monitoring as part of the intervention rather than as an adherence monitoring tool for evaluation, directly observed therapy (which could include partner or video observation), changes in scheduling for medications and dispensation (e.g. moving to a three- or six-month drug supply), regimen simplification, addressing food insecurity through supplementation or support, or task shifting. The ‘other’ intervention component code could be applied to provide added nuance to one of the a priori codes or to characterize any active intervention component not in the original list of strategies.

**Outcomes** (measures, efficacy, and comparison condition) included any adherence-related outcome reported and viral outcomes specifically when available. For multiple adherence or VL outcomes, codes captured the primary outcome when stated. To account for the wide range of study designs (e.g. from pilot to cluster randomized trials) and outcome measures (e.g. multiple operationalizations of adherence and/or inclusion of VL) outcomes were characterized by coders as having *“any evidence of effect”* for studies reporting any promise, having some signal of impact, or positive trends (regardless of sample size, power, or reaching significance). *“Statistically significant effect”* defined only those studies that reported statistically significant (*p* ≤ .05) intervention effects on one or more adherence outcome (including VL). Studies could encompass multiple intervention strategies and evaluations, so intervention characteristics and outcomes were extracted separately for all active arms in a given study. No additional calculations were used in this review to determine efficacy or effect size.

The extracted data were combined, cleaned, and examined quantitatively using Excel and SPSS software. Simple descriptive statistics were used to describe the study information and adherence interventions, including overall and grouped frequencies (k) and proportions, as well as medians and interquartile ranges (IQR) to describe the distribution on non-normally distributed information. Selected intervention strategies that demonstrated impact on outcomes, particularly VL outcomes, were examined qualitatively and described in-depth.

## Results

**Fig. 1:**
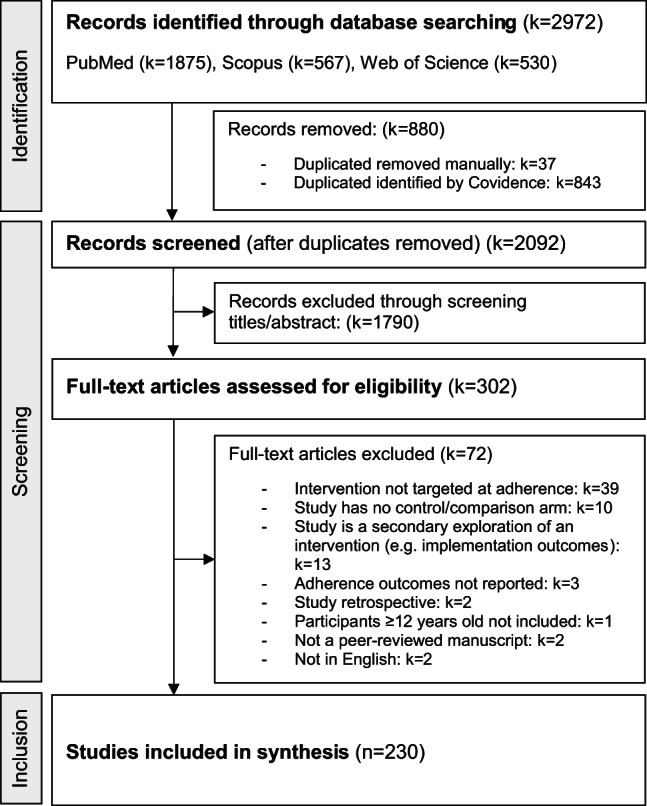
Preferred Reporting Items for Systematic reviews and Meta-Analyses (PRISMA) flow diagram for the systematic review detailing the database searches, the number of abstracts screened and the full texts retrieved

A total of 230 published studies (Supplementary material File C) containing evaluations of 262 separate interventions with anticipated effects on ART adherence (as primary, secondary or exploratory objectives) were included (see Fig. 1).

### Description of Included Studies

#### Year of Publication and Implementation

An average of 22 studies were published per year in the five years from 2015 to 2019; and 30 per year from 2020 to 2023. In 2024, only three publications were included given the timing of the literature review. The year when study implementation started was extracted when available (k = 198/230, 86%) and ranged from 2003 to 2022. Nearly half were started before 2015 (k = 94/198, 48%) or began between 2015 and 2020 (k = 96/198, 48%). The period between start of study recruitment and publication date ranged from 1 to 13 years (median 5 years, IQR 3–6).

#### Study Design, Power, and Sample Size

Nearly all studies used random assignment to the intervention (k = 215/230, 94%). Single arm, pre/post designs were uncommon (k = 7/230, 3%), as were quasi-experimental designs (k = 8/230, 4%). An increase in cluster-randomized trials was noted over time; the 30 cluster randomized trials that were included were published largely over the last three years and nearly exclusively involved sites in Africa (k = 27/30, 90% of cluster randomized trials). Pilot studies, as self-described in articles, made up over a quarter of the included evaluations (k = 66/230, 29%).

A total of 102 (44% of the 230 studies) studies contained clear statements related to statistical power to detect efficacy of the intervention(s) on the primary adherence outcome(s). Combining these with the studies that appeared likely to have adequate power to detect intervention effects, based on sample size (e.g. sample sizes ≥ 300) and design features (e.g. single arm intervention evaluated with intent to treat), most were likely adequately powered to detect medium to large intervention effects (k = 122/230, 56%). Among studies reported as *not* being a pilot studies (k = 164), power was estimated as sufficient for 74% (K = 121/164).

Sample sizes ranged from six to 16,208. For studies other than cluster randomized trials, the median sample size was 144 (IQR 63–341), and for cluster randomized trials 699 (IQR 358–1302) participants. Pilot studies had a median sample size of 53 (IQR 33–90). Across all included studies, a total of 97,037 PWH were represented.

#### Location

**Fig. 2:**
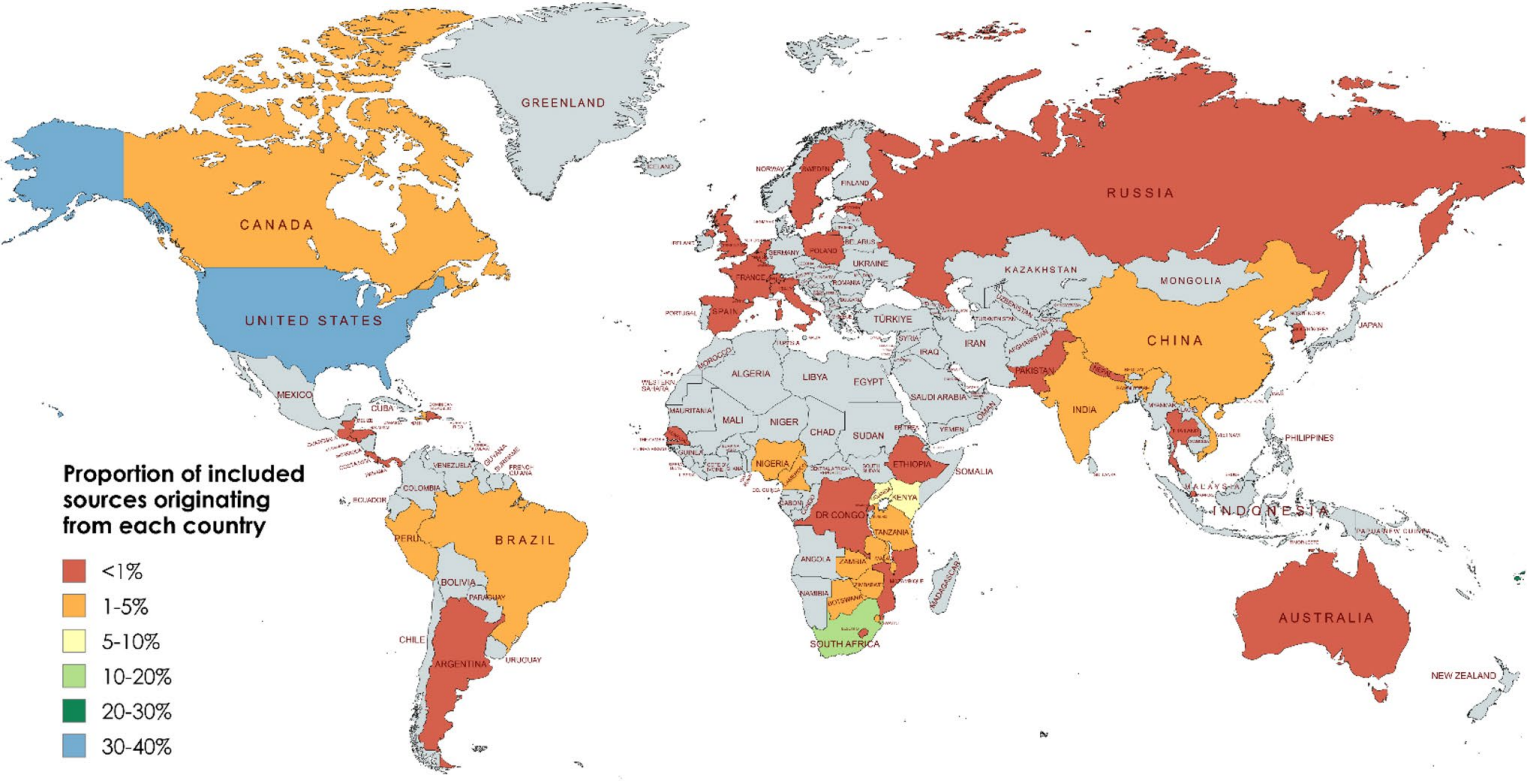
Global distribution of countries in which the research included in this review was conducted (image created with MapChart.net) [19]

As depicted in the map in Fig. 2 [19], studies involving PWH in Africa and North America dominated the evidence base (k = 106/230, 46% and k = 80/230, 35%, respectively). Studies included participants from 17 countries in Africa, with two countries heavily represented: individuals in South Africa and Uganda participated in 24% (26/106) and 21% (22/106) of studies, respectively. The United States accounted for most of the studies conducted in North America (k = 77/81, 96%). Studies engaging participants from Asia (k = 25/230, 11%) included eight countries, with participants in China (k = 8/25, 32%) and India (k = 6/25, 24%) making the largest contributions. Studies with PWH residing in Central and South America, Australia, and Europe cumulatively represented less than 10% (k = 19/230, 8%) of the evidence base.

#### Populations of Focus

Nearly half (k = 102/230, 44%) of all studies evaluated interventions with general HIV-positive clinic populations. Youth and adolescents (participants aged 12 to 24) were generally well represented (k = 51/230, 22%), more so over the last five years, with most (k = 35/51, 69%) youth-inclusive studies published after 2019. Other populations of interest were: people who use substances (k = 29/230, 13%), postpartum and pregnant women (k = 23/230, 10%), and people with mental health challenges, such as depression (k = 22/230, 10%). Several populations were less well represented, including men who have sex with men (k = 14/230, 6%), specific races or ethnicities (k = 9/230, 4%), PWH engaged with the justice system (k = 9/230, 4%), those struggling with food insecurity or poverty (k = 9/230, 4%), transgender PWH (k = 3/230, 1%), hospitalized patients (k = 2/230, 1%), and people engaged with sex-work (k = 2/230, 1%).

By location, youth- and adolescent-focused interventions were predominantly conducted in Africa (k = 31/51, 61%), as were all studies focused on postpartum and pregnant women (k = 23/23, 100%) and people engaged with sex work (k = 2/2, 100%). All interventions for incarcerated or PWH engaged with the justice system (k = 9/9, 100%), and the majority of interventions with inclusion criteria focusing on transgender individuals (k = 2/3, 67%) or people who use substances (k = 17/29, 59%), were conducted in North America.

#### ART-Related Inclusion Criteria

Studies showed substantial variability in inclusion and exclusion criteria in terms of ART exposure and experiences. The majority of studies recruited treatment experienced individuals (k = 118/230, 51%) versus those initiating treatment for the first time (k = 32/230, 14%), while the remainder recruited mixed samples (k = 19/230, 8%) or did not specify treatment history (k = 61/230, 27%). Nearly a fifth (k = 44/230, 19%) focused on PWH with known adherence challenges, and 17% (k = 39/230) used viral non-suppression as an inclusion criterion. Studies published from 2020 onwards used viral non-suppression as an inclusion criterion (k = 26/122, 21%) more frequently than those published earlier (k = 13/108, 12%). Nearly half described ‘other’ inclusion criteria (k = 108/230, 47%), such as area of residence (k = 5/230, 2% used rural residence) or access to internet or other technology (k = 12/230, 5%).

### Outcome Measures and Timing

Nearly all studies used multiple adherence measures, but most adopted the clinical trial approach of selecting a single outcome measure at a single time point to evaluate efficacy. Longitudinal modeling of all available timepoints was uncommon. The time from baseline to final outcome ranged from one month to 48 months, with a median of six months (IQR 6–12). Studies reported as pilot studies used outcomes closer to baseline (median six months, IQR 3–6) than non-pilot studies (median 12 months, IQR 6–12).

**Fig. 3:**
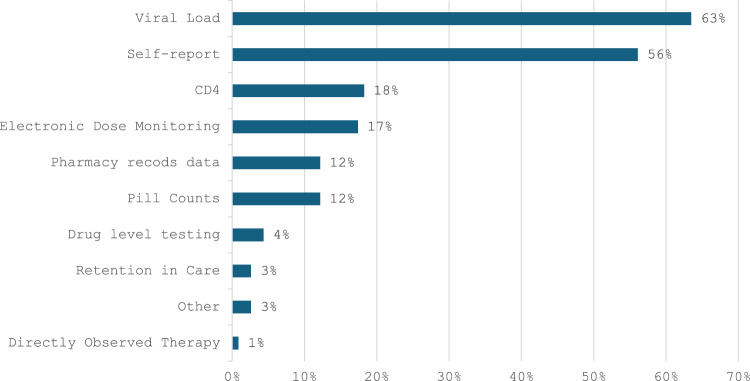
Measures of treatment success and adherence used by studies in this review

Across all studies (Fig. 3), the most common measure used in estimating or characterizing the effect of intervention(s) was VL (k = 146/230, 64%), a quarter of which were pilot studies (k = 35/146, 24%). Time from baseline to VL outcome ranged from 2.5 months to 48 months, with a median of 12 months (IQR 6–12). Among studies with information about the threshold for suppression (k = 117), use of 200 copies/mL was used as the threshold in 63% of studies (k = 74/117), and 50 copies/mL in 42% (k = 49/117).

Self-reported adherence was also a common outcome; a majority (k = 129/230, 56%) of studies included a self-reported measure, most commonly asking participants to estimate percent adherence over a given time period (k = 45/129, 35%), often on a Visual Analogue Scale or a single item question. The Adult AIDS Clinical Trials Group (AACTG) adherence instrument was used in 10% of the studies (k = 13/129) [20] while fewer studies used recall of daily dose taking (k = 7/129, 5%), an approach embedded in the AACTG. Recall of doses missed versus taken, appeared in 8% of the studies (k = 10/129). Reliance solely on self-reported adherence was characteristic of a minority (k = 36/230, 16%) of studies (eight of these were self-described as pilots).

### Interventions

Two-hundred and sixty-two active interventions were evaluated across the 230 published studies. The vast majority of studies evaluated a single intervention (k = 204/230, 89%), while 9% (k = 20/230) evaluated two and 3% (k = 6/230) evaluated three active interventions in a single study.

**Fig. 4:**
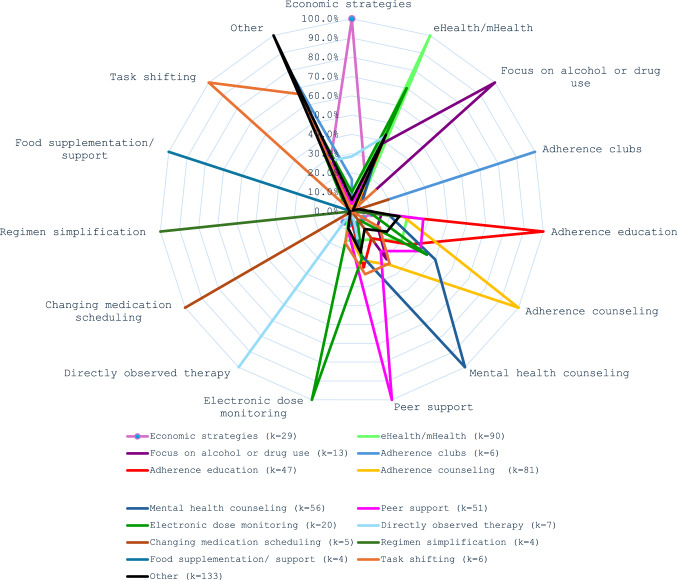
Overlap of intervention strategies evaluated in this review

The 14 a priori intervention strategy codes captured one or more intervention features in most of the active interventions - only 10% (k = 26/262) were coded as only ‘other’ for their intervention approach. Among the coded intervention characteristics (see Table 2), the most common strategies evaluated were eHealth/ mHealth approaches (k = 90/262, 34%), followed by adherence counseling (k = 81/262, 31%). Most interventions used a combination of intervention strategies (k = 182/262, 70%). There was substantial overlap in strategies (see Fig. 4), particularly for education, adherence counselling, counselling for mental health and wellness, and the use of peers. 80% of the interventions assigned to the ‘other’ category overlapped with additional a priori intervention strategies (107/133 categorized as ‘other’).

**Table 2:** Intervention components included in this review (K, %), by those for which there was some evidence of support reported for the full intervention (k, %) and by use of viral load (VL) as an outcome and evidence of support

Intervention Strategy	Study arms with a specific intervention strategy		Any Adherence Outcome				VL Outcome					
			Any evidence of effect for the intervention strategy		Significant impact on any adherence outcome		Had a VL outcome measure		Any evidence of effect for the intervention strategy		Significant impact on a VL outcome	
As a proportion of:	All arms (k=262)		Arms using the specified strategy						Arms with a VL outcome			
	k	%	k	%	k	%	k	%	k	%	k	%
eHealth/ mHealth	90	34%	55	61%	28	31%	53	59%	27	51%	13	25%
Adherence counseling	81	31%	57	70%	31	38%	52	64%	31	60%	15	29%
Adherence education	64	24%	36	56%	23	36%	36	56%	16	44%	7	19%
Mental health and well-being counseling	56	21%	35	63%	16	29%	33	59%	19	58%	8	24%
Peer support	51	19%	32	63%	21	41%	34	67%	15	44%	9	26%
Economic strategies	29	11%	23	79%	15	52%	21	72%	17	81%	10	48%
Electronic dose monitoring as an intervention	20	8%	16	80%	7	35%	15	75%	7	47%	1	7%
Focus on alcohol or drug use	13	5%	7	54%	5	38%	12	92%	6	50%	4	33%
Directly observed therapy	7	3%	3	43%	2	29%	4	57%	1	25%	0	0%
Adherence clubs	6	2%	3	50%	3	50%	6	100%	3	50%	2	33%
Regimen simplification	6	2%	3	50%	1	17%	6	100%	2	33%	0	0%
Task shifting	6	2%	5	83%	5	83%	5	83%	4	80%	3	60%
Changing medication scheduling	5	2%	4	80%	3	60%	5	100%	4	80%	3	60%
Food supplementation or support	4	2%	3	75%	2	50%	1	25%	1	100%	0	0%
Other strategies	133	51%	81	61%	54	41%	75	56%	36	48%	21	28%
Only ‘other’ strategies	26	10%	15	58%	12	46%	12	46%	5	42%	4	33%

*k* number, % percentage, *VL* viral load, significance as ≤0.05

More than half the interventions (k = 159/262, 61%) had some evidence of effect on any adherence-related outcome. When limited to results reaching significance in favor of the intervention on any adherence-related outcome, this dropped to 37% (k = 98/262). Among the 166 interventions reporting VL outcomes, 52% (k = 87/166) reported some evidence of effect for the intervention. Fewer studies reported significant effects on VL outcomes (k = 47/166, 28%). Restricting this to only the 98 interventions evaluating viral outcomes with sufficient power, the proportion reporting significant outcomes was similar (k = 28/98, 29%). Table 2 summarizes the proportions of studies using each intervention strategy that had some measure of effect and that had significant evidence of VL improvement.

### eHealth and mHealth Strategies

Interventions employing eHealth/ mHealth-based strategies represented 34% of the studies included in this review (k = 90/262). Variability in the specific tools and features being evaluated reflect an evolution of intervention capabilities over time; overall there was some evidence of effect reported in 66% of studies evaluating eHealth/ mHealth approaches (k = 55/90). Recent interventions using eHealth/ mHealth-tethered real-time monitoring and text-based interactions [21, 22], offered interactive, tailored text messages (versus non-interactive reminders) [23], used various media platforms to promote engagement [24], and involved complete web-based intervention packages [25]. The majority of interventions involving eHealth (k = 60/90, 79%%) were *not* pilot studies and a quarter (k = 13/53, 25%) of those evaluated with VL outcomes demonstrated significant evidence of effect for the intervention approach.

#### Adherence-Focused Counseling

Adherence-focused counseling is distinguished from adherence education, which provides didactic information and is increasingly the standard of care at ART initiation in many countries. Adherence-focused counseling, included in 31% of the interventions evaluated (k = 81/262), was characterized as specific counseling packages or approaches developed for the purposes of improving adherence, dominated by cognitive behavioral therapy (CBT) approaches [26–39], but also included motivational interviewing (MI) [39–48], and problem-solving counseling tailored to barriers to adherence [49–53]. These had a pre-determined number of sessions, training material, and, in many cases, systems of supervision for the interventionists (often peers or community health workers). Some found success in targeted counseling for people with alcohol or substance use disorders specifically [32, 54], in an effort to improve adherence. Others used eHealth/ mHealth platforms to deliver counseling sessions [32, 55]. Over half of the interventions that included adherence-focused counseling as a component had some evidence of effect (k = 57/81, 70%) and a third of the studies that included a VL outcome demonstrated significant impact of the intervention (k = 15/52, 29%).

#### Mental Health-Focused Counseling

Counseling focused on mental health specifically was represented in 21% (k = 56/262) of the interventions. Distinguished from adherence education sessions and adherence-focused counseling, these interventions targeted aspects of mental health and well-being as a means to improve adherence through enhanced overall quality of life, adjustment, and relief from mental health symptoms. Using evidence-based, mental health therapy approaches (e.g. CBT) or adaptations of such strategies for lay person implementation, counseling interventions were delivered with a variety of additional features (e.g. individuals [31, 56, 57], groups [58–62], in-community [63, 64], and by peers [65, 66]) and for a range of conditions (including depression [26, 67, 68], anxiety [28, 69], trauma [70], body-image disorder [27], and bipolar mood disorder [57]). They also ranged in dosage from brief interventions of one session [68, 71], to regular sessions over one to three months [65, 72, 73]. Combining therapy with antidepressant treatment was included in one study [74], and phone delivery of mental health counseling was also evaluated [75], although effects on adherence did not differ between groups in either study. Counseling focused on mental health and well-being had evidence of effect on one or more adherence outcome in 63% (k = 35/56) and had significant impact on VL in about a quarter (k = 8/33, 24%) of the studies that included VL evaluation. Of note, two thirds (k = 38/56, 68%) of these studies were published after 2019, suggesting this approach is growing in popularity.

#### Social Determinants of Health Outcomes

Efforts to evaluate the impact of interventions focused on the elimination or reduction of social challenges to health outcomes, such as food insecurity and poverty, were sparce. Although food security-focused interventions were uncommon (k = 4/262, 1%), three quarters (k = 3/4, 75%) had some evidence of effect on adherence. The two studies showing significant impact were targeted at people with some level of food insecurity rather than the general population [76, 77]. 

Finance-based interventions were more common, represented in 11% (k = 29/262) of the interventions. Economic strategies were most commonly described as performance-based behavioral incentives (k = 19/29, 66%) or aimed to address poverty as a social determinant of health through microfinancing, unconditional cash transfers, or the provision of work resources (k = 10/29, 34%). The results of these studies suggest that giving people a choice over their incentive goals can be impactful in improving adherence [78], and smartphone-based incentives could be effective in improving suppression [79]. Others, like the Shamba Maisha intervention [80], focused on improving poverty as a social determinant of health by providing equipment (water pumps) in addition to microfinance loans, which successfully improved VL outcomes. These overwhelmingly showed some degree of intervention effect (k = 23/29, 79%), and when evaluating VL, demonstrated some evidence of effect in 81% (k = 17/21), and significant impact on VL in 48% (k = 10/21). This impact has the potential to be sustained longer term: the Suubi plus Adherence family-based economic empowerment intervention demonstrated a positive impact over 48 weeks [81, 82]. 

Some of the intervention strategies identified in the ‘other’ category included education specifically focused on nutrition [83] or financial accounting and planning [81, 84, 85], to support the primary strategy of food supplementation or economic incentives, all with some evidence of success.

#### Service-Level Interventions

Few interventions targeted systems-level change. Task-shifting was used in only 2% (k = 6/262) of active interventions, demonstrating some efficacy in 83% (k = 5/6) and a significant impact on VL in 60% (3/5 with VL outcomes). Successful task-shifting included changes in delivery of VL testing [86], psychotherapy or counseling [40, 72, 87], and clinical care [88]. A change in medication scheduling (k = 5/262, 2%) was similarly effective, with all using VL as an outcome: the strategy had a significant impact in 60% (k = 3/5) and some evidence of effect in 80% (k = 4/5). As shown in Table 2, regimen simplification (k = 6/262, 2%) had mixed findings (k = 3/6, 50% with some evidence). Service-level strategies included in the ‘other’ category covered *where* care took place, such as in the community or at home (k = 20/133, 15% of the ‘other’ interventions) and *how* care was given, such as in a caregiver-child dyad, with a partner, or with a treatment supporter (k = 8/133, 6%). They also included changes in *when* care was provided, such as same-day results for VL or CD4 testing (k = 4/133, 3%). Others with some evidence of effect on adherence included active tracking and outreach for missed visits [89], workflow modification [90], supervision of community healthcare workers [91], and facility-level training [92]. 

#### Other Strategies

As indicated in Table 2, ‘other’ strategies were noted for over half the sample (k = 133/262, 51%), although the majority of these studies (k = 107/133, 80%) also used strategies in the a-priori defined intervention list. Exploration of text within this category identified a number of intervention features not well represented in our a-priori extraction list, the most common being adherence reminders (k = 39/133, 29%). Three studies examined the impact of yoga as a wellbeing intervention to improve adherence, but only one showed a significant impact on VL outcomes [93]. Self-help booklets, instrumental support or help with transport, referrals, and case management were also described.

## Discussion

This review identified a large number of interventional studies that evaluated a wide range of adherence intervention strategies with mixed success on adherence outcomes, including VL suppression. Our review indicates that an increasing number of well-structured studies are focused on improving adherence to ART. There has also been a shift towards the use of objective measurements of adherence as research outcomes, most commonly an HIV-1 VL, as used by 64% of the studies reviewed. This likely reflects focus on the population-based UNAIDS 95-95-95 goal, which has prompted countries to track and invest in promoting viral suppression in 95% of those taking ART.

The vast majority of studies were conducted in Africa and North America (mostly the United States). This reflects the global burden of disease: southern and eastern Africa bear the highest rates of HIV in the world, and are where more than two thirds of global new HIV infections occur annually [93–96]. The geographic spread of included evidence may also reflect research funding access, as most international funding for HIV programs and research traditionally came from the United States, e.g. the National Institutes for Health (NIH), United States President’s Emergency Plan for AIDS Relief (PEPFAR) and US contributions to the Global Fund to Fight AIDS, Tuberculosis and Malaria [10]. This distribution may change as geopolitics shift global funding for HIV research and program delivery. Notably, the two countries with the highest HIV prevalence in the world, Eswatini and Lesotho [97], made up less than 1% of included studies in this review.

Nearly half of the studies examined adherence interventions in a general clinic population. Although certain ‘key populations’, including men who have sex with men, people who use substances, people in prisons, sex workers, and transgender people are considered particularly vulnerable to systemic and social barriers challenging ART adherence across all contexts [10, 98], the bulk of the epidemic in southern and eastern Africa affects the general population [99]. Interventions that are scalable to the whole population may be particularly appropriate in generalized epidemics, and thus need to be tested in these contexts. However, in addition to this work and the emphasis on youth and prevention of mother-to-child transmission, the increasing burden on key populations in other regions, including Asia, e.g. transgender people and sex workers, highlights the urgency for better representation in ART adherence research.

In some settings, two-thirds of people starting treatment are restarting ART rather than initiating treatment for the first time [100], often after substantial treatment interruptions accompanied by a decline in CD4 count and health status [101]. In fact, the proportion of those starting treatment with a CD4 count below 50 that are treatment experienced has increased from 14 to 57% in just 10 years [102]. Opportunistic infections and other acute symptoms are a common reason for re-engagement with services [103]. In this review, only two studies explicitly included hospitalized individuals [56, 104], in an era when advanced HIV and admission may be crucial routes to re-entry for those with adherence challenges.

This review identified a wide range of adherence intervention strategies, including adherence education and counseling, mental health-focused counseling, eHealth/ mHealth technologies, peer-based support and/or use of peers for intervention delivery, electronic dose monitoring support, supplementary treatment or counseling for alcohol or drug use, incentivizing or simplifying treatment access, and food supplementation, with widely varying rates of success in achieving viral suppression both between strategies and within categories across different studies. The most commonly researched adherence interventions, namely adherence-focused counseling and eHealth/ mHealth approaches, achieved a significant reduction in VL in less than a third of the studies. A number of other less-thoroughly researched interventions show promise in achieving viral suppression and indicate important mechanisms of behavior change. Micro-finance and food security address social and structural determinants of health but largely at the individual level. A systems-level approach could be more impactful by removing some of these barriers (e.g. extreme poverty or food insecurity) altogether, thereby reducing the burden and personal responsibility of engagement for PWH. Addressing social determinants of health like poverty and food insecurity were the focus of very few studies, though most (79% of economic strategies and 75% of food support) demonstrated some evidence of effect on adherence. Improving systems of care to be more person-centered also showed promise and is recommended by the World Health Organization [105, 106]. This may include reducing the frequency of refills, reorganizing work flows, task shifting care, point of care VL monitoring, community services, and services for caregiver-child or partner dyads. Such approaches may be particularly important in high burden settings in Africa, where the bulk of people affected by HIV are the ‘general population’ and facilities provide care for high volumes of PWH with increasingly limited resources [98]. 

ART remains lifelong treatment, and PWH will continue to need to take it as they age through different phases of life [107, 108]. Although many studies showcase adherence interventions which improved rates of viral suppression, our review found that this benefit was measured at a median of less than a year post-intervention. When considering future research, exploration of long-term outcomes for successful adherence interventions, perhaps to three to five years, must be prioritized. Additionally, only a few studies examined the sustained impact of the intervention over time. Understanding the pattern of effect over multiple time points is critical to understanding what strategies may work for whom, at different points in their lifelong relationship with ART [109–112]. 

Lastly, it is unlikely that a particular strategy will work for every person living with HIV, and even for a particular individual at all points in their life’s journey on ART [113]. The factors influencing engagement shift and change over time, tipping people towards or away from engagement at different times in their lives; [6] therefore, service delivery needs to respond to this complex and dynamic interplay of influential factors. This review describes a range of interventions with evidence of effect on adherence and treatment outcomes. However, none were found to work consistently across all studies and contexts. Therefore, the next step in refining the response to the HIV epidemic is to understand what to offer to whom, and at what time [114, 115]. Taking individual preferences into consideration by giving people a choice between a range of options is one way of matching interventions to those for whom they may work best [116, 117], and this shared decision-making can improve health outcomes [118, 119]. Offering a choice needs options, of which we have many. However, guiding this choice is challenging, and requires further research to explore for whom and how the interventions work.

### Strengths and Limitations

This review included a large number of sources and drew on a wide range of evidence, including controlled trials, to describe the range of interventions that have been tested to improve and support adherence to ART across the world. Similar systematic reviews completed in 2011, 2014, and 2017, include only 27 [13], 85 [12], and 86 [11] studies, respectively; our review included 230 studies. We were also able to report on a large number of studies that used objective measures of adherence and treatment success to describe the impact of interventions. However, important limitations are that the review was conducted rapidly and with a large team. While measures were put in place to mitigate bias, such as double screening and regular discussion of the application of eligibility, the criteria may have been applied slightly differently by different team members. Furthermore, evidence was not assessed for risk of bias. While the rapid turnaround may have introduced errors in screening and extraction, the large number of included sources help to triangulate findings and reduce bias. Finally, our hybrid systematic-narrative review coded effective interventions on the basis of reported findings in each study, rather than calculating effect sizes that could be compared as a common metric across studies. Our results are presented as an overview of the literature rather than a rigorous assessment of the strength of the evidence.

## Conclusions

Much research has been conducted in the past decade to improve adherence to ART with only limited impact on the critical measure of ART success: viral suppression. Encouraging adherence to oral ART remains crucial to individual health and program success in the face of highly limited access to long-acting injectable forms of treatment. The impact of counseling and educational methods on adherence is limited, similar to the low impact of the many eHealth/ mHealth support options assessed in recent years. The future research agenda should continue to explore broader measures to address the underlying social determinants of health and to reduce persistent barriers to sustainably accessing care, in addition to behavioral interventions targeted at individuals with HIV. Offering the right interventions at the right time to the groups of people who may benefit the most will also require more understanding of how and why different interventions work for different people, to support the more person-centered care that will likely be needed to achieve global 95-95-95 goals.

## Supplementary Information

Below is the link to the electronic supplementary material.

Supplementary Material 1

Supplementary Material 2

Supplementary Material 3

## Data Availability

Data is available on reasonable request to the corresponding author.
