# Supplementary material for: Antiretroviral Therapy Adherence Interventions in the Era of Universal Test and Treat: A Hybrid Systematic-Narrative Literature Review of Global Evidence
Source: AIDS Behav. 2025 Oct 6;30(1):291–306. doi: 10.1007/s10461-025-04867-9 (PMC12816096; doi:10.1007/s10461-025-04867-9)
Supplement: Supplementary file 1 — Supplementary Material 1 [file 10461_2025_4867_MOESM1_ESM.pdf]

Supplementary material File A. Search terms used for each database

Database	Search strategy
PubMed	(HIV[MeSH Terms] OR (HIV[Title/Abstract]) OR (Human Immunodeficiency Virus[Title/Abstract]) OR (Anti-Retroviral Agents[MeSH Terms]) OR (antiretroviral therapy, highly active[MeSH Terms]) OR (Antiretroviral[Title/Abstract]) OR (HAART[Title/Abstract]) OR (ART[Title/Abstract]) OR (ARVs[Title/Abstract]))<br><br>AND<br><br>(Medication adherence[MeSH Terms] OR (patient compliance[MeSH Terms]) OR (Treatment adherence and compliance[MeSH Terms]) OR (adherence[Title/Abstract]) OR (compliance[Title/Abstract]) OR (nonadherence[Title/Abstract]) OR (non-compliance[Title/Abstract]) OR (non-adherence[Title/Abstract]) OR (noncompliance[Title/Abstract]) OR (Drug monitoring[Title/Abstract]) OR (drug monitoring[Title/Abstract]) OR (medication monitoring[Title/Abstract]) OR (medication persistence[Title/Abstract]))<br><br>AND<br><br>(clinical trials, randomized[MeSH Terms] OR (controlled clinical trials, randomized[MeSH Terms]) OR (randomized controlled trial[Title/Abstract]) OR (randomised controlled trial[Title/Abstract]) OR (intervention[Title/Abstract]))<br><br>AND<br><br>(viral load[MeSH Terms] OR (antigen, cd4[MeSH Terms]) OR (viral suppression[Title/Abstract]) OR (CD4[Title/Abstract]) OR (biological outcome[Title/Abstract]) OR (biological outcomes[Title/Abstract]) OR (clinical outcome[Title/Abstract]) OR (clinical outcomes[Title/Abstract]))<br><br>NOT (protocol[Title])<br><br>NOT (predictors[Title/Abstract])
Scopus	(TITLE-ABS-KEY (("acquired immune-deficiency syndrome*" OR "acquired immuno-deficiency syndrome*" OR "acquired immunodeficiency syndrome*" OR "human immune-deficiency virus" OR "human immuno-deficiency virus" OR "human immunodeficiency virus*" OR hiv-1* OR hiv-2* OR hiv OR hiv1 OR hiv2) AND ("anti-hiv" OR "anti-retroviral" OR antiretroviral OR art OR arvs OR cart OR haart) AND ("drug monitoring" OR "medication monitoring" OR "medication persistence" OR adherence OR adherent OR compliance OR compliant OR non-adherence OR non-adherent OR non-compliance OR non-compliant OR nonadherence OR nonadherent OR noncompliance OR noncompliant)) AND TITLE (((clinical AND trial) OR random*))) AND PUBYEAR > 2014
Web of Science	(("acquired immune-deficiency syndrome*" OR "acquired immuno-deficiency syndrome*" OR "acquired immunodeficiency syndrome*" OR "human immune-deficiency virus" OR "human immuno-deficiency virus" OR "human immunodeficiency virus*" OR hiv-1* OR hiv-2* OR hiv OR hiv1 OR hiv2) AND ("anti-hiv" OR "anti-retroviral" OR antiretroviral OR art OR arvs OR cart OR haart) AND ("drug monitoring" OR "medication monitoring" OR "medication persistence" OR adherence OR adherent OR compliance OR compliant OR non-adherence OR non-adherent OR non-compliance OR non-compliant OR nonadherence OR nonadherent OR noncompliance OR noncompliant)) (Topic) and ((clinical AND trial) OR random*) (Title)
