# Supplementary material for: Antiretroviral Therapy Adherence Interventions in the Era of Universal Test and Treat: A Hybrid Systematic-Narrative Literature Review of Global Evidence
Source: AIDS Behav. 2025 Oct 6;30(1):291–306. doi: 10.1007/s10461-025-04867-9 (PMC12816096; doi:10.1007/s10461-025-04867-9)
Supplement: Supplementary file 2 — Supplementary Material 2 [file 10461_2025_4867_MOESM2_ESM.pdf]

Supplementary material File B: Covidence data extraction template

General information

First author's last name and date of publication

Primary Outcome of Study

Note primary outcome of study (may not be adherence)

Characteristics of included studies

Study design

- Randomised controlled trial (has a control condition)
- Non-randomised experimental study (quaziexperimental, no random assignment, has control/comparison condition)
- Prospective observational study (pre/post; within subject)
- Other

Is the study a pilot?

- Yes
- No

Adherence in the study

- Primary focus (clear articulated link between intervention and adherence improvement)
- Secondary focus (link between intervention and adherence improvement noted as secondary)
- Tertiary (link between intervention and adherence improvement is only implied)

Describe adherence outcomes and data collection

Start year of data collection/data collected in the study

Select all adherence measures included in the study outcomes

- ☐ Self-report
- ☐ Pill-count
- ☐ Pharmacy refill
- ☐ Electronic dose monitoring
- ☐ DOT (in person)
- ☐ Video DOT
- ☐ Real time adherence self report (e.g., mobile app, texting)
- ☐ CD4
- ☐ Viral load/Suppression/Viral outcome
- ☐ Drug levels (DBS, Plasma, Urine, Hair)
- ☐ Other

Select the Primary adherence outcome

- ○ Self-report
- ○ Pill-count
- ○ Pharmacy refill
- ○ Electronic dose monitoring
- ○ DOT (in person)
- ○ Video DOT
- ○ Real time adherence self report (e.g., mobile app, texting)
- ○ CD4
- ○ Viral load/suppression
- ○ Drug levels (DBS, Plasma, Urine, Hair)

Notes on primary adherence outcome

Name, type, cut-off used

Month (on study) adherence primary outcome was assessed

- ○ 1 month
- ○ 3 months
- ○ 6 months
- ○ 12 months
- ○ 18 months
- ○ 24 months
- ○ Other

Sufficient power (80% or higher) to detect effect on adherence primary outcome?

- ○ Yes
- ○ No
- ○ Not stated

Intervention impact on primary adherence outcome based on analysis presented?

- ○ Intervention sig better
- ○ Intervention better (ns)
- ○ Same (no difference)
- ○ Control better (sig or otherwise)
- ○ Other

Secondary adherence outcome

- ○ Self report
- ○ Pill count
- ○ Pharmacy refill
- ○ Electronic dose montoring
- ○ DOT (in person)
- ○ video DOT
- ○ Real time self report (mobile app, texting)
- ○ CD4
- ○ Viral load/suppression
- ○ Drug level (DBS, Plasma, Urine, Hair)
- ○ NONE
- ○ Other

Notes on secondary adherence outcome

Provide cut-off (threshold for success) used for primary adherence outcomes (if used)- NA if outcome is continuous.

Month for secondary adherence outcome

- ○ 1 month
- ○ 3 months
- ○ 6 months
- ○ 12 months
- ○ 18 months
- ○ 24 months
- ○ Other

Sufficient power (80% or higher) to detect effect on secondary adherence outcome?

- ○ Yes
- ○ No
- ○ Not stated

Intervention impact on second adherence outcome based on analysis presented?

- ○ Intervention sig better
- ○ Intervention better (ns)
- ○ Same
- ○ Control better (sig or otherwise)
- ○ Other

Are there additional adherence outcomes not captured above?

- ○ Yes
- ○ No

Additional outcomes of interest that are not included above

List any other outcomes for support/lack of support of intervention that should be considered but are not in outcomes 1, 2 or 3

Population(s)

Country/Countries included in research population

Continents where study was conducted

- ☐ Africa
- ☐ Asia
- ☐ Australia
- ☐ Europe
- ☐ North America
- ☐ South America
- ☐ Other

Reported Population(s) included

Only tic boxes for those identities/groups that are specifically listed/reported. Otherwise select gen clinic sample.

- ☐ Pregnant/post-partum
- ☐ Youth/Adol
- ☐ Hospitalized
- ☐ GBMSM
- ☐ TGW
- ☐ PW use substances
- ☐ Sex Work engaged
- ☐ Incarcerated/recent release
- ☐ Unspecified/general clinic sample
- ☐ Other

Inclusion criteria (must be...)

Artificial (clinical trial setting with retention strategies)

- ☐ Treatment experienced
- ☐ New to treatment
- ☐ Viremic
- ☐ Virally suppressed
- ☐ People with adherence problems
- ☐ History of missed clinic visits/retention problems
- ☐ Other

Total number of participants enrolled

Notes on participant retention or engagement impacting generalizability

Any peculiarities in retention or strategies used to retain participants that would limit generalizability

INTERVENTION(s)

Number of active interventions

Presently the form is set for 1 active intervention

- ○ 1
- ○ 2
- ○ 3
- ○ Other

Intervention Description Open Text (one line description)

Include key characteristics of intervention- we will create code list from these

Features of Intervention 1 (select all)

- ☐ eHealth/mobile health (apps, texts)
- ☐ dose monitoring device(s)
- ☐ DOT/vDOT
- ☐ economic incentives/micro finance
- ☐ adherence clubs
- ☐ peer support (adherence supporter, peer counselor)
- ☐ task shifting (nurse vs md care, peer vs psychologist care)
- ☐ counseling support- mental health focused (coping, trauma, depression)
- ☐ adherence focused counseling (problem solving, MI on adherence)
- ☐ educational counseling- adherence training
- ☐ regimen simplification (reduced frequency or co-formulation to reduce number of pills)
- ☐ alcohol/drug use treatments
- ☐ food/nutrition supplements
- ☐ change in dispensing schedule (frequency of clinic attendance)
- ☐ Other

Intervention 1 Duration/Dose

Weeks duration
Number of sessions
Length of sessions

Explain any additional interventions evaluated in the study

CONTROL CONDITIONS

Control/comparison Description

Features of Comparison Condiction (select all/any adherence support provided in the comparison condiction)

- [ ] eHealth/mobile health (apps, texts) related to adherence
- [ ] Dose monitoring device
- [ ] DOT/vDOT
- [ ] economic incentives/micro finance
- [ ] adherence clubs
- [ ] peer support (adherence supporter, peer counselor)
- [ ] task shifting (nurse vs md care, peer vs psychologist care)
- [ ] counseling support- mental health focused (coping, trauma, depression)
- [ ] adherence focused counseling (problem solving, MI on adherence)
- [ ] educational counseling- adherence training
- [ ] regimen simplification (reduced frequency or co-formulation to reduce number of pills)
- [ ] alcohol/drug use treatments
- [ ] food/nutrition supplements
- [ ] change in dispensing schedule (frequency of clinic attendance)
- [ ] Other

Open Notes
