# Supplementary material for: Antiretroviral Therapy Adherence Interventions in the Era of Universal Test and Treat: A Hybrid Systematic-Narrative Literature Review of Global Evidence
Source: AIDS Behav. 2025 Oct 6;30(1):291–306. doi: 10.1007/s10461-025-04867-9 (PMC12816096; doi:10.1007/s10461-025-04867-9)
Supplement: Supplementary file 3 — Supplementary Material 3 [file 10461_2025_4867_MOESM3_ESM.pdf]

Supplementary material File C. Studies included in hybrid-narrative review[1-231]

STUDY FEATURES											INTERVENTON STRATEGIES																OUTCOMES			
First Author and date	Intervention*	Design	Pilot	N	Powered	SR Only	Has VL	Location(s)	ART Status	General	Economic strategies	eHealth/ mHealth	Alcohol/drug use	Adherence clubs	Adherence education	Adherence counseling	MH counseling	Peer support	Electronic dose monitoring	Directly observed therapy	Medication scheduling	Regimen simplification	Food support	Task shifting	Other	**Total Strategies**	Any evidence of effect	Significant impact	Any evidence of effect: VL	Significant impact: VL
**Abas 2018**	1	RCT	1	32	0	0	1	Africa	Unclear	1	0	0	0	0	0	1	1	0	0	0	0	0	0	0	0	2	1	0	1	0
**Abbas 2023**	1	RCT	0	126	1	1	0	Asia	Unclear	1	0	0	0	0	0	0	1	0	0	0	0	0	0	0	0	1	1	1		
**Abdulrahman 2017**	1	RCT	0	242	1	0	1	Asia	ART naïve	1	0	1	0	0	0	0	0	1	0	0	0	0	0	0	1	3	1	1	1	1
**Abiodun 2021**	1	RCT	0	212	1	0	1	Africa	ART Hx	0	0	1	0	0	0	0	0	0	0	0	0	0	0	0	1	2	1	1	1	1
**Abuogi 2022**	1	RCT	0	1338	1	1	0	Africa	Unclear	0	0	1	0	0	1	0	0	0	0	0	0	0	0	0	1	3	0	0		
	2	RCT	0			1	0				0	1	0	0	0	0	0	0	0	0	0	0	0	0	1	2	0	0		
	3	RCT	0			1	0				0	1	0	0	1	0	0	1	0	0	0	0	0	0	1	4	0	0		
**Alsan 2017**	1	RCT	1	40	0	0	1	North America	Unclear	1	1	0	0	0	0	0	0	0	0	0	0	0	0	0	0	1	1	0	1	0
	2	RCT	1			0	1				1	0	0	0	0	0	0	0	0	0	0	0	0	0	1	2	1	1	1	1
**Amico 2022**	1	RCT	0	89	0	0	1	North America	Unclear	0	0	1	0	0	0	1	0	0	1	0	0	0	0	0	1	4	1	0	0	0
**Amone 2023**	1	RCT	0	540	1	0	1	Africa	ART naïve	0	1	0	0	1	0	0	0	1	0	0	0	0	0	0	1	4	0	0	0	0

Supplementary material File C. Studies included in hybrid-narrative review[1-231]

STUDY FEATURES											INTERVENTON STRATEGIES																OUTCOMES			
First Author and date	Intervention*	Design	Pilot	N	Powered	SR Only	Has VL	Location(s)	ART Status	General	Economic strategies	eHealth/ mHealth	Alcohol/drug use	Adherence clubs	Adherence education	Adherence counseling	MH counseling	Peer support	Electronic dose monitoring	Directly observed therapy	Medication scheduling	Regimen simplification	Food support	Task shifting	Other	**Total Strategies**	Any evidence of effect	Significant impact	Any evidence of effect: VL	Significant impact: VL
**Ashburn 2021**	1	RCT	0	379	0	0	1	Africa	Unclear	0	0	0	0	0	0	0	0	0	0	0	0	0	0	0	1	1	0	0	0	0
**Attonito 2020**	1	RCT	0	243	0	1	1	North America	Unclear	0	0	0	0	0	1	0	1	1	0	0	0	0	0	0	0	3	1	0	1	0
**Aunon 2023**	1	RCT	0	119	0	0	1	Africa	ART naïve	0	0	1	0	0	1	0	0	0	0	0	0	0	0	0	1	3	1	0	1	0
**Ayer 2021**	1	RCT	0	468	1	0	0	Asia	ART Hx	1	0	1	0	0	0	0	0	0	0	0	0	0	0	0	1	2	0	0		
**Ayieko 2024**	1	RCT	1	201	1	0	1	Africa	Unclear	1	0	0	0	0	0	0	0	0	0	0	0	0	0	0	1	1	1	1	0	0
**Barnabas 2022**	1	RCT	0	173	1	0	1	Africa	Mixed	1	0	0	0	0	0	0	0	0	0	0	1	0	0	0	1	2	1	1	1	1
**Bellagamba 2019**	1	RCT	0	197	1	0	1	Europe	ART Hx	1	0	0	0	0	0	0	0	0	0	0	0	1	0	0	0	1	0	0	0	0
**Benzekri 2019**	1	RCT	1	26	0	0	0	Africa	Unclear	0	0	0	0	0	0	0	0	0	0	0	0	0	1	0	0	1	0	0		
**Bermudez 2018**	1	RCT	0	702	1	0	1	Africa	ART Hx	0	1	0	0	0	0	0	0	0	0	0	0	0	0	0	0	1	1	0	1	0
**Bien-Gund 2021**	1	RCT	1	29	0	0	1	North America	Unclear	1	1	0	0	0	0	0	0	0	1	0	0	0	0	0	0	2	1	0	1	0
**Blashill 2017**	1	RCT	1	44	0	0	0	North America	ART Hx	0	0	0	0	0	0	1	1	0	1	0	0	0	0	0	1	4	1	1		

Supplementary material File C. Studies included in hybrid-narrative review[1-231]

STUDY FEATURES											INTERVENTON STRATEGIES															OUTCOMES			
First Author and date	Intervention*	Design	Pilot	N	Powered	SR Only	Has VL	Location(s)	ART Status	General	Economic strategies	eHealth/ mHealth	Alcohol/drug use	Adherence clubs	Adherence education	Adherence counseling	MH counseling	Peer support	Electronic dose monitoring	Directly observed therapy	Medication scheduling	Regimen simplification	Food support	Task shifting	Other	**Total Strategies**	Any evidence of effect	Significant impact	Any evidence of effect: VL	Significant impact: VL
**Bogart 2017**	1	RCT	0	215	1	0	0	North America	ART Hx	0	0	0	0	0	1	1	0	0	0	0	0	0	0	0	0	2	1	1		
**Bogart 2021**	1	RCT	1	76	0	0	0	North America	Unclear	0	0	0	0	0	1	1	1	0	0	0	0	0	0	0	0	3	1	0		
**Bogart 2023**	1	RCT	0	166	0	0	1	North America	ART Hx	0	0	0	0	0	0	1	0	1	1	0	0	0	0	1	1	5	1	1	1	0
**Bouris 2017**	1	RCT	1	106	0	0	1	North America	ART Hx	0	0	0	0	1	0	1	0	0	0	0	0	0	0	0	1	3	1	1	1	1
**Brandt 2019**	1	RCT	1	27	0	1	0	North America	Unclear	0	0	0	0	0	0	0	1	0	0	0	0	0	0	0	0	1	0	0		
**Broughton 2016**	1	RCT	0	100	1	0	0	Africa	ART Hx	1	0	0	0	0	0	0	0	0	0	0	0	0	0	0	1	1	1	1		
**Byonanebye 2021**	1	RCT	0	600	1	0	1	Africa	Mixed	1	0	1	0	0	1	0	0	0	0	0	0	0	0	0	1	3	0	0	0	0
**Cabral 2018**	1	RCT	0	348	1	0	1	North America	ART Hx	0	0	0	0	0	1	1	0	1	0	0	0	0	0	0	1	4	0	0	0	0
**Calder 2020**	1	RCT	0	210	0	1	0	Africa	ART naïve	0	0	0	0	0	0	0	0	1	0	0	0	0	0	1	1	3	1	1		
**Carey 2020**	1	RCT	1	42	0	0	1	North America	Unclear	0	0	0	0	0	0	0	1	0	0	0	0	0	0	0	1	2	1	0	1	0
**Chang 2021**	1	RCT	0	2148	1	0	1	Africa	Unclear	0	0	1	0	0	0	1	0	0	0	0	0	0	0	0	1	3	1	1	0	0

Supplementary material File C. Studies included in hybrid-narrative review[1-231]

STUDY FEATURES											INTERVENTON STRATEGIES																OUTCOMES			
First Author and date	Intervention*	Design	Pilot	N	Powered	SR Only	Has VL	Location(s)	ART Status	General	Economic strategies	eHealth/ mHealth	Alcohol/drug use	Adherence clubs	Adherence education	Adherence counseling	MH counseling	Peer support	Electronic dose monitoring	Directly observed therapy	Medication scheduling	Regimen simplification	Food support	Task shifting	Other	**Total Strategies**	Any evidence of effect	Significant impact	Any evidence of effect: VL	Significant impact: VL
**Chang 2023**	1	RCT	0	541	1	0	1	Africa	ART naïve	0	0	0	0	0	1	0	0	0	0	0	0	0	0	0	0	1	0	0	0	0
**Chatha 2020**	1	RCT	0	100	1	0	0	Asia	ART Hx	1	0	0	0	0	1	1	0	0	0	0	0	0	0	0	0	2	1	1		
**Chattopadhyay 2017**	1	RCT	0	60	0	0	0	Asia	ART Hx	1	0	0	0	0	1	1	0	0	0	0	0	0	0	0	1	3	1	1		
**Chawana 2017**	1	RCT	0	50	0	0	1	Africa	Unclear	0	0	1	0	0	0	0	0	0	0	1	0	0	0	0	0	2	1	1	1	0
**Christodoulou 2020**	1	RCT	1	28	0	0	1	North America	ART Hx	0	0	0	0	0	1	0	0	0	0	0	0	0	0	0	1	2	0	0	0	0
**Christopoulos 2018**	1	RCT	0	230	1	0	1	North America	Unclear	1	0	1	0	0	0	0	0	0	0	0	0	0	0	0	1	2	1	0	1	0
**Cohen 2022**	1	RCT	0	720	1	0	1	Africa	ART Hx	1	1	0	0	0	0	0	0	0	0	0	0	0	0	0	0	1	0	0	0	0
**Coker 2015**	1	RCT	0	600	1	0	1	Africa	ART naïve	0	0	1	0	0	0	0	1	1	0	0	0	0	0	0	1	4	0	0	0	0
	2	RCT	0			0	1				0	1	0	0	0	0	1	1	0	0	0	0	0	0	1	4	0	0	0	0
**Cook 2019**	1	RCT	0	182	1	0	1	North America	Unclear	0	0	0	1	0	0	0	0	0	0	0	0	0	0	0	0	1	0	0	0	0
**CorreiaLima 2023**	1	RCT	0	144	1	1	0	South America	ART Hx	1	0	0	0	0	0	0	1	0	0	0	0	0	0	0	1	2	1	1		

Supplementary material File C. Studies included in hybrid-narrative review[1-231]

STUDY FEATURES											INTERVENTON STRATEGIES																OUTCOMES			
First Author and date	Intervention*	Design	Pilot	N	Powered	SR Only	Has VL	Location(s)	ART Status	General	Economic strategies	eHealth/ mHealth	Alcohol/drug use	Adherence clubs	Adherence education	Adherence counseling	MH counseling	Peer support	Electronic dose monitoring	Directly observed therapy	Medication scheduling	Regimen simplification	Food support	Task shifting	Other	**Total Strategies**	Any evidence of effect	Significant impact	Any evidence of effect: VL	Significant impact: VL
**Cote 2020**	1	RCT	0	88	1	1	0	North America	ART Hx	1	0	1	0	0	1	0	0	0	0	0	0	0	0	0	0	2	1	0		
**Crable 2021**	1	RCT	0	68	0	1	0	North America	Unclear	0	0	0	0	0	0	0	0	0	0	0	0	0	0	0	1	1	0	0		
**Cunningham 2018**	1	RCT	0	356	1	0	1	North America	ART Hx	0	0	0	0	0	0	0	0	1	0	0	0	0	0	0	1	2	1	1	1	1
**Cunningham 2020**	1	RCT	0	73	0	0	1	North America	ART Hx	0	1	0	0	0	0	0	0	0	0	0	0	0	0	0	0	1	1	0	1	0
**Cuong 2016**	1	RCT	0	640	1	0	1	Asia	ART naïve	1	0	0	0	0	0	0	0	1	0	0	0	0	0	0	1	2	0	0	0	0
**deBruin 2017**	1	RCT	0	224	1	0	1	Europe	Mixed	1	0	0	0	0	0	1	0	0	1	0	0	0	0	0	0	2	1	1	1	1
**DeFulio 2021**	1	RCT	1	51	0	0	0	North America	ART Hx	0	1	1	0	0	0	0	0	0	0	1	0	0	0	0	0	3	1	1		
**Denison 2020**	1	RCT	0	273	1	0	1	Africa	ART Hx	0	0	0	0	0	0	0	0	1	0	0	0	0	0	0	0	1	1	0	0	0
**Denison 2022**	1	RCT	0	100	0	0	1	Africa	ART Hx	0	0	0	0	0	1	0	0	1	0	0	0	0	0	0	1	3	0	0	0	0
**Derose 2023**	1	RCT	1	115	0	0	1	North America	ART Hx	1	0	0	0	0	0	0	0	0	0	0	0	0	1	0	1	2	1	0	1	0
**DiPrete 2019**	1	RCT	0	381	1	0	1	North America	ART Hx	0	0	0	0	0	0	1	1	0	0	0	0	0	0	0	1	3	0	0	0	0

Supplementary material File C. Studies included in hybrid-narrative review[1-231]

STUDY FEATURES											INTERVENTON STRATEGIES																OUTCOMES			
First Author and date	Intervention*	Design	Pilot	N	Powered	SR Only	Has VL	Location(s)	ART Status	General	Economic strategies	eHealth/ mHealth	Alcohol/drug use	Adherence clubs	Adherence education	Adherence counseling	MH counseling	Peer support	Electronic dose monitoring	Directly observed therapy	Medication scheduling	Regimen simplification	Food support	Task shifting	Other	**Total Strategies**	Any evidence of effect	Significant impact	Any evidence of effect: VL	Significant impact: VL
**Diress 2020**	1	Pre Post Single Arm	0	235	0	0	1	Africa	ART Hx	1	0	0	0	0	1	0	0	0	0	0	0	0	0	0	1	2	1	1	1	1
**Doerfler 2016**	1	RCT	1	33	0	0	1	North America	ART Hx	1	0	0	0	0	0	0	1	0	0	0	0	0	0	0	0	1	0	0	0	0
**Donenberg 2022**	1	RCT	0	356	1	0	1	Africa	ART Hx	0	0	0	0	0	1	1	1	0	0	0	0	0	0	0	0	3	0	0	0	0
**Dow 2020**	1	RCT	1	105	0	0	1	Africa	ART Hx	0	0	0	0	0	0	0	1	0	0	0	0	0	0	0	0	1	1	0	1	0
**Drain 2020**	1	RCT	0	390	1	0	1	Africa	ART Hx	1	0	0	0	0	0	0	0	0	0	0	0	0	0	1	1	2	1	1	1	1
**Dulli 2020**	1	RCT	0	349	1	1	0	Africa	ART Hx	0	0	1	0	0	1	0	0	1	0	0	0	0	0	0	1	4	0	0		
**Edelman 2019**	1	RCT	0	51	0	0	1	Other: Not stated	ART Hx	0	0	0	1	0	0	0	0	0	0	0	0	0	0	0	0	1	0	0	0	0
**Ekwunife 2022**	1	RCT	0	246	1	0	1	Africa	ART Hx	0	1	0	0	0	0	1	0	0	0	0	0	0	0	0	0	2	1	0	1	0
**El-Sadr 2017**	1	RCT	0	16208	1	0	1	North America	ART Hx	1	1	0	0	0	0	0	0	0	0	0	0	0	0	0	0	1	1	1	1	1
**Ellsworth 2021**	1	RCT	1	63	0	0	1	North America	Unclear	1	0	1	0	0	0	0	0	0	1	0	0	0	0	0	0	2	0	0	0	0
**Enriquez 2015**	1	RCT	1	20	0	0	1	North America	ART Hx	1	0	0	0	0	0	1	0	1	0	0	0	0	0	0	0	2	1	1	1	1

Supplementary material File C. Studies included in hybrid-narrative review[1-231]

STUDY FEATURES											INTERVENTON STRATEGIES															OUTCOMES			
First Author and date	Intervention*	Design	Pilot	N	Powered	SR Only	Has VL	Location(s)	ART Status	General	Economic strategies	eHealth/ mHealth	Alcohol/drug use	Adherence clubs	Adherence education	Adherence counseling	MH counseling	Peer support	Electronic dose monitoring	Directly observed therapy	Medication scheduling	Regimen simplification	Food support	Task shifting	Other	**Total Strategies**	Any evidence of effect	Significant impact	Any evidence of effect: VL	Significant impact: VL
**Enriquez 2019**	1	RCT	1	30	0	0	1	North America	ART Hx	1	0	0	0	0	0	1	0	1	0	0	0	0	0	0	0	2	1	1	1	1
**Eron 2019**	1	RCT	0	1141	1	0	1	Europe; North America	ART Hx	1	0	0	0	0	0	0	0	0	0	0	0	1	0	0	0	1	0	0	0	0
**Fahey 2020**	1	RCT	0	530	1	0	1	Africa	ART naïve	1	1	1	0	0	0	0	0	0	0	0	0	0	0	0	0	2	1	1	1	1
	2	RCT	0			0	1				1	1	0	0	0	0	0	0	0	0	0	0	0	0	0	2	1	1	1	1
**Fan 2023**	1	RCT	0	344	1	1	0	Asia	ART naïve	0	0	1	0	0	1	0	0	0	0	0	0	0	0	0	1	3	1	0		
**Ferrand 2017**	1	RCT	0	166	0	0	1	Africa	ART naïve	0	0	0	0	0	0	0	0	1	0	0	0	0	0	0	0	1	1	1	1	1
**Ferraris 2023**	1	RCT	1	60	0	0	0	Africa	ART Hx	1	0	0	0	0	0	0	0	0	0	0	0	0	0	0	1	1	1	1		
**Fiscella 2018**	1	RCT	0	360	1	0	1	North America	ART Hx	1	0	0	0	0	0	1	0	0	0	0	0	0	0	0	0	1	0	0	0	0
**Fox 2018**	1	RCT	0	1266	1	0	1	Africa	Unclear	1	0	0	0	0	0	0	0	1	0	0	0	0	0	0	1	2	0	0	0	0
**Garofalo 2016**	1	RCT	1	105	0	0	1	North America	Unclear	0	0	1	0	0	0	0	0	0	0	0	0	0	0	0	1	2	0	0	0	0
**Giordano 2016**	1	RCT	0	460	1	0	1	North America	Unclear	0	0	0	0	0	0	1	0	1	0	0	0	0	0	0	0	2	0	0	0	0

Supplementary material File C. Studies included in hybrid-narrative review[1-231]

STUDY FEATURES											INTERVENTON STRATEGIES																OUTCOMES			
First Author and date	Intervention*	Design	Pilot	N	Powered	SR Only	Has VL	Location(s)	ART Status	General	Economic strategies	eHealth/ mHealth	Alcohol/drug use	Adherence clubs	Adherence education	Adherence counseling	MH counseling	Peer support	Electronic dose monitoring	Directly observed therapy	Medication scheduling	Regimen simplification	Food support	Task shifting	Other	**Total Strategies**	Any evidence of effect	Significant impact	Any evidence of effect: VL	Significant impact: VL
**Giovenco 2024**	1	RCT	1	215	0	0	1	Africa	ART Hx	0	0	0	0	0	0	0	0	0	0	0	0	0	0	0	1	1	1	0	1	0
	2	RCT	1			0	1				0	1	0	0	0	0	0	0	0	0	0	0	0	0	1	2	1	0	1	0
**Glasner 2020**	1	RCT	1	35	0	0	1	Other: Not stated. Unclear without investigating urther outside the paper (OASIS clinic)	Unclear	0	0	1	0	0	0	1	0	0	0	0	0	0	0	0	0	2	1	1	1	1
**Glasner 2022**	1	RCT	1	54	0	0	1	Other: Not stated. Unclear without investigating urther outside the paper (OASIS clinic)	Unclear	0	0	1	1	0	0	1	0	0	0	0	0	0	0	0	0	3	1	1	1	1
**Go 2020**	1	RCT	0	440	1	0	1	Asia	ART Hx	0	0	0	1	0	0	0	1	0	0	0	0	0	0	0	0	2	1	0	1	0
	2	RCT	0			0	1				0	1	1	0	0	0	1	0	0	0	0	0	0	0	0	3	1	1	1	1
**Gonzales 2022**	1	RCT	0	156	1	0	1	South America	ART naïve	0	0	0	1	0	0	0	1	0	0	0	0	0	0	0	0	2	0	0	0	0
**Goodrich 2021**	1	RCT	0	420	1	0	1	Africa	ART Hx	1	0	0	1	0	0	0	0	0	0	0	0	0	0	1	0	2	0	0	0	0
**Graham 2020**	1	RCT	1	60	0	0	1	Africa	Mixed	0	0	0	0	0	0	1	0	1	1	0	0	0	0	0	0	3	1	0	1	0

Supplementary material File C. Studies included in hybrid-narrative review[1-231]

STUDY FEATURES											INTERVENTON STRATEGIES																OUTCOMES			
First Author and date	Intervention*	Design	Pilot	N	Powered	SR Only	Has VL	Location(s)	ART Status	General	Economic strategies	eHealth/ mHealth	Alcohol/drug use	Adherence clubs	Adherence education	Adherence counseling	MH counseling	Peer support	Electronic dose monitoring	Directly observed therapy	Medication scheduling	Regimen simplification	Food support	Task shifting	Other	**Total Strategies**	Any evidence of effect	Significant impact	Any evidence of effect: VL	Significant impact: VL
**Gross 2015**	1	RCT	0	259	1	0	1	Africa; North America; South America	Unclear	1	0	0	0	0	0	0	0	0	0	1	0	0	0	0	0	1	0	0	0	0
**Gross 2019**	1	RCT	0	521	1	0	1	Africa; Asia; North America; South America	ART Hx	1	0	1	0	0	0	1	0	0	0	0	0	0	0	0	1	3	1	0	1	0
**Guo 2018**	1	RCT	1	62	0	0	0	Asia	ART naïve	1	0	1	0	0	1	0	0	0	0	0	0	0	0	0	0	2	0	0		
**Gwadz 2015**	1	RCT	1	95	0	0	1	North America	Unclear	1	0	0	0	0	0	1	0	1	0	0	0	0	0	0	0	2	1	1	1	1
**Haas 2023**	1	RCT	0	516	1	0	0	Africa	Unclear	1	0	0	0	0	0	1	0	1	0	0	0	0	0	0	0	2	1	0		
**Haberer 2016**	1	RCT	1	62	0	0	1	Africa	ART naïve	1	0	1	0	0	0	0	0	1	1	0	0	0	0	0	1	4	1	1	0	0
	2	RCT	1			0	1				0	1	0	0	0	0	0	1	1	0	0	0	0	0	1	4	0	0	0	0
**Han 2020**	1	RCT	1	20	0	0	0	Asia	ART Hx	1	0	0	0	0	0	1	1	0	0	0	0	0	0	0	0	2	0	0		
**Han 2021a**	1	RCT	0	140	1	0	0	Asia	ART Hx	1	0	0	0	0	0	1	1	0	0	0	0	0	0	0	0	2	1	0		
**Han 2021b**	1	RCT	0	61	1	1	0	Asia	ART Hx	1	0	1	0	0	1	1	1	1	0	0	0	0	0	0	0	5	0	0		
**Hightow-Weidman 2021**	1	RCT	0	146	1	0	1	North America	Unclear	0	1	1	0	0	1	0	0	0	0	0	0	0	0	0	1	4	1	1	1	1

Supplementary material File C. Studies included in hybrid-narrative review[1-231]

STUDY FEATURES											INTERVENTON STRATEGIES																OUTCOMES			
First Author and date	Intervention*	Design	Pilot	N	Powered	SR Only	Has VL	Location(s)	ART Status	General	Economic strategies	eHealth/ mHealth	Alcohol/drug use	Adherence clubs	Adherence education	Adherence counseling	MH counseling	Peer support	Electronic dose monitoring	Directly observed therapy	Medication scheduling	Regimen simplification	Food support	Task shifting	Other	**Total Strategies**	Any evidence of effect	Significant impact	Any evidence of effect: VL	Significant impact: VL
**Himelhoch 2017**	1	RCT	1	30	0	0	0	North America	ART Hx	0	0	1	0	0	0	0	0	0	0	0	0	0	0	0	1	2	0	0		
**Horvath 2019**	1	RCT	1	90	0	1	0	North America	Unclear	0	0	1	0	0	1	0	0	0	0	0	0	0	0	0	1	3	0	0		
**Hosek 2018**	1	RCT	0	103	0	0	1	North America	ART naïve	0	0	0	0	0	1	1	0	0	0	0	0	0	0	0	0	2	1	1	1	1
**Huhn 2015**	1	RCT	0	60	1	0	1	North America	ART Hx	1	0	0	0	0	0	0	0	0	0	0	1	0	0	0	0	1	1	0	1	0
**in'tVeld 2019**	1	RCT	0	560	1	0	1	Africa	Unclear	1	0	0	0	0	0	0	1	0	0	0	0	0	0	0	0	1	0	0	0	0
**Ingerski 2021**	1	RCT	1	32	0	0	1	North America	ART naïve	0	0	0	0	0	0	1	0	0	0	0	0	0	0	0	0	1	1	1	1	1
**Ingersoll 2015**	1	RCT	1	63	0	0	0	North America	ART Hx	0	0	1	0	0	0	0	0	0	0	0	0	0	0	0	1	2	1	1		
**Jackson 2021**	1	RCT	0	206	0	1	1	Africa	ART Hx	0	0	1	0	0	1	0	0	0	0	0	0	0	0	0	0	2	0	0	0	0
**Jenewari 2021**	1	RCT	0	225	1	1	0	Africa	ART Hx	1	0	0	0	0	0	1	0	0	0	0	0	0	0	0	1	2	1	1		
	2	RCT	0			1	0				0	0	0	0	0	0	0	0	0	0	0	0	0	0	1	2	0	0		
	3	RCT	0			1	0				0	0	0	0	0	1	0	0	0	0	0	0	0	0	0	2	0	0		

Supplementary material File C. Studies included in hybrid-narrative review[1-231]

STUDY FEATURES											INTERVENTON STRATEGIES																OUTCOMES			
First Author and date	Intervention*	Design	Pilot	N	Powered	SR Only	Has VL	Location(s)	ART Status	General	Economic strategies	eHealth/ mHealth	Alcohol/drug use	Adherence clubs	Adherence education	Adherence counseling	MH counseling	Peer support	Electronic dose monitoring	Directly observed therapy	Medication scheduling	Regimen simplification	Food support	Task shifting	Other	**Total Strategies**	Any evidence of effect	Significant impact	Any evidence of effect: VL	Significant impact: VL
**Jiao 2022**	1	RCT	0	576	1	0	1	Asia	ART Hx	0	0	1	0	0	1	0	0	1	0	0	0	0	0	0	1	4	1	1	0	0
**Jones 2016**	1	RCT	1	120	0	0	1	South America	Unclear	1	0	0	0	0	1	1	0	1	0	0	0	0	0	0	0	3	1	1	1	1
	2	RCT	1			0	1				0	0	0	0	1	0	0	0	0	0	0	0	0	0	1	2	1	1	1	1
	3	RCT	1			0	1				0	0	0	0	1	1	0	1	0	0	0	0	0	0	1	2	1	0	1	0
**Jones 2021**	1	RCT	0	1399	1	0	0	Africa	ART Hx	0	0	0	0	0	1	0	0	0	0	0	0	0	0	0	0	1	0	0		
**JonesASK 2019**	1	RCT	0	111	0	0	1	Africa	Unclear	1	0	0	0	0	1	0	0	0	0	0	0	0	0	0	0	1	0	0	0	0
**Junkins 2021**	1	RCT	1		0	0	1	North America	ART Hx	0	0	1	0	0	0	1	1	0	0	0	0	0	0	0	0	3	1	0	1	0
**Kaihin 2015**	1	Quasi	1	46	0	0	0	Asia	ART Hx	0	0	0	0	0	0	0	0	0	0	0	0	0	0	0	1	1	1	1		
**Kalichman 2016**	1	RCT	0	600	1	0	1	North America	ART Hx	1	0	0	0	0	0	1	0	0	0	0	0	0	0	0	0	1	1	1	1	1
	2	RCT	0			0	1				0	1	0	0	0	0	0	0	0	0	0	0	0	0	1	2	0	0	0	0
	3	RCT	0			0	1				0	1	0	0	0	1	0	0	0	0	0	0	0	0	1	1	0	0	0	0

Supplementary material File C. Studies included in hybrid-narrative review[1-231]

STUDY FEATURES											INTERVENTON STRATEGIES																OUTCOMES			
First Author and date	Intervention*	Design	Pilot	N	Powered	SR Only	Has VL	Location(s)	ART Status	General	Economic strategies	eHealth/ mHealth	Alcohol/drug use	Adherence clubs	Adherence education	Adherence counseling	MH counseling	Peer support	Electronic dose monitoring	Directly observed therapy	Medication scheduling	Regimen simplification	Food support	Task shifting	Other	**Total Strategies**	Any evidence of effect	Significant impact	Any evidence of effect: VL	Significant impact: VL
**Kalichman 2019**	1	RCT	1	50	0	1	0	Africa	Unclear	1	0	0	0	0	0	0	0	0	0	0	0	0	0	0	1	1	1	1		
**Kalichman 2021**	1	RCT	0	251	0	0	1	North America	Unclear	1	0	0	0	0	0	0	1	0	0	0	0	0	0	0	0	1	0	0	0	0
**Kalichman 2022**	1	RCT	0	240	1	0	1	North America	Unclear	1	0	1	0	0	0	0	1	0	0	0	0	0	0	0	0	2	0	0	0	0
	2	RCT	0			0	1				0	0	0	0	0	0	1	0	0	0	0	0	0	0	0	1	0	0	0	0
**Kalichman 2023**	1	Quasi	0	435	1	0	1	North America	ART naïve	1	0	0	0	0	0	1	0	0	0	0	0	0	0	0	0	1	0	0	0	0
**Kibu 2022**	1	RCT	0	210	0	1	0	Africa	ART Hx	1	0	1	0	0	0	0	0	0	0	0	0	0	0	0	1	2	0	0		
	2	RCT	0			1	0				0	1	0	0	0	0	0	0	0	0	0	0	0	0	0	1	0	0		
**Kim 2019**	1	RCT	1	306	1	0	0	Africa	ART naïve	0	0	1	0	0	0	0	0	0	0	0	0	0	0	0	1	2	1	1		
**King 2017**	1	Pre Post Single Arm	0	85	1	0	1	North America	ART Hx	0	0	1	0	0	0	0	0	0	0	0	0	0	0	0	1	2	1	1	1	1
**Kinuthia 2021**	1	RCT	0	824	1	0	1	Africa	Mixed	0	0	1	0	0	1	0	0	0	0	0	0	0	0	0	1	3	0	0	0	0
	2	RCT	0			0	1				0	1	0	0	0	0	0	0	0	0	0	0	0	0	0	1	0	0	0	0

Supplementary material File C. Studies included in hybrid-narrative review[1-231]

STUDY FEATURES											INTERVENTON STRATEGIES																OUTCOMES			
First Author and date	Intervention*	Design	Pilot	N	Powered	SR Only	Has VL	Location(s)	ART Status	General	Economic strategies	eHealth/ mHealth	Alcohol/drug use	Adherence clubs	Adherence education	Adherence counseling	MH counseling	Peer support	Electronic dose monitoring	Directly observed therapy	Medication scheduling	Regimen simplification	Food support	Task shifting	Other	**Total Strategies**	Any evidence of effect	Significant impact	Any evidence of effect: VL	Significant impact: VL
**Kizito 2023**	1	RCT	1	89	0	0	0	Africa	ART Hx	0	0	0	0	0	0	1	0	0	0	0	0	0	0	0	1	1	1	1		
	2	RCT	1			0	0				0	0	0	0	0	0	1	1	0	0	0	0	0	0	1	3	1	1		
**Kizito 2024**	1	RCT	0	702	1	0	0	Africa	Unclear	0	1	0	0	0	0	0	0	0	0	0	0	0	0	0	1	2	1	0		
**Klein 2022**	1	RCT	0	25	0	0	1	North America	Unclear	1	0	0	0	0	1	0	0	0	0	0	0	1	0	0	1	3	1	0	1	0
**Kopo 2023**	1	RCT	0	307	1	0	1	Africa	ART Hx	0	0	1	0	0	0	0	1	0	0	0	1	0	0	0	0	3	1	0	1	0
**Korthuis 2021**	1	RCT	0	281	1	0	1	Asia	ART Hx	1	0	0	0	0	0	0	0	0	0	1	0	0	0	0	0	1	0	0	0	0
**Krupitsky 2019**	1	RCT	0	200	1	0	1	Europe	Mixed	0	0	0	1	0	0	0	0	0	0	0	0	0	0	0	0	1	1	1	1	1
**Kuo 2019**	1	RCT	1	110	0	0	1	North America	ART Hx	0	0	1	0	0	0	1	0	0	0	0	0	0	0	0	0	2	1	0	1	0
**Kurth 2016**	1	RCT	0	494	1	0	1	North America	ART Hx	0	0	1	0	0	0	1	1	0	0	0	0	0	0	0	1	4	1	0	1	0
**Kurth 2019**	1	RCT	0	236	1	0	1	Africa	ART Hx	1	0	1	0	0	0	0	0	0	0	0	0	0	0	0	1	2	0	0	0	0
**Larson 2023**	1	RCT	0	363	1	0	0	Africa	Mixed	0	0	0	0	0	1	0	0	1	0	0	0	0	0	0	0	2	0	0		

Supplementary material File C. Studies included in hybrid-narrative review[1-231]

STUDY FEATURES											INTERVENTON STRATEGIES																OUTCOMES			
First Author and date	Intervention*	Design	Pilot	N	Powered	SR Only	Has VL	Location(s)	ART Status	General	Economic strategies	eHealth/ mHealth	Alcohol/drug use	Adherence clubs	Adherence education	Adherence counseling	MH counseling	Peer support	Electronic dose monitoring	Directly observed therapy	Medication scheduling	Regimen simplification	Food support	Task shifting	Other	**Total Strategies**	Any evidence of effect	Significant impact	Any evidence of effect: VL	Significant impact: VL
**Limbada 2022**	1	RCT	0	1393	1	0	1	Africa	ART Hx	1	0	0	0	0	0	0	0	0	0	0	0	0	0	0	1	1	1	0	1	1
	2	RCT	0			0	1				0	0	0	1	0	0	0	0	0	0	0	0	0	0	0	1	1	1	1	0
**Linnemayr 2017a**	1	RCT	0	155	1	0	0	Africa	ART Hx	1	1	0	0	0	0	0	0	0	1	0	0	0	0	0	0	2	1	1		
**Linnemayr 2017b**	1	RCT	0	332	1	0	0	Africa	ART Hx	0	0	1	0	0	0	0	0	0	0	0	0	0	0	0	1	2	0	0		
	2	RCT	0			0	0				0	1	0	0	0	0	0	0	0	0	0	0	0	0	1	2	0	0		
**Liu 2022**	1	RCT	0	112	1	0	1	North America	Unclear	1	0	1	0	0	0	0	0	0	1	0	0	0	0	0	1	3	1	0	1	0
**Lyatuu 2022**	1	RCT	0	2637	1	0	1	Africa	Mixed	0	1	0	0	0	0	0	0	1	0	0	0	0	0	0	1	3	1	1	0	0
**MacCarthy 2020**	1	RCT	1	179	0	0	0	Africa	ART Hx	0	0	1	0	0	0	0	0	0	1	0	0	0	0	0	1	3	0	0		
	2	RCT	1			0	0				0	1	0	0	0	0	0	1	1	0	0	0	0	0	0	3	1	0		
**Mageda 2023**	1	RCT	0	120	1	0	1	Africa	ART Hx	0	0	0	0	0	0	1	1	0	0	0	0	0	0	0	0	2	1	1	1	1
**Magidson 2021**	1	RCT	1		0	0	0	Africa	ART Hx	0	0	0	0	0	0	1	1	1	0	0	0	0	0	0	0	3	1	1		

Supplementary material File C. Studies included in hybrid-narrative review[1-231]

STUDY FEATURES										INTERVENTON STRATEGIES																OUTCOMES			
First Author and date	Intervention*	Design	Pilot	N	Powered	SR Only	Has VL	Location(s)	ART Status	General	Economic strategies	eHealth/ mHealth	Alcohol/drug use	Adherence clubs	Adherence education	Adherence counseling	MH counseling	Peer support	Electronic dose monitoring	Directly observed therapy	Medication scheduling	Regimen simplification	Food support	Task shifting	Other	**Total Strategies**	Any evidence of effect	Significant impact	Any evidence of effect: VL	Significant impact: VL
**Magidson 2022**	1	RCT	0	61	1	1	0	North America	ART Hx	0	0	0	0	0	0	1	1	0	0	0	0	0	0	0	1	3	0	0		
**Mao 2018**	1	RCT	0	98	0	1	0	Australia	ART Hx	1	0	1	0	0	0	0	0	0	0	0	0	0	0	0	1	2	1	0		
**Mavhu 2020**	1	RCT	0	500	1	0	1	Africa	Mixed	0	0	1	0	0	0	1	1	1	0	0	0	0	0	0	0	4	1	1	1	1
**McCoy 2016**	1	RCT	0	805	1	0	0	Africa	ART naïve	0	1	0	0	0	0	0	0	0	0	0	0	0	0	0	0	1	1	1		
**McCoy 2017a**	1	RCT	0	805	1	0	0	Africa	ART naïve	1	1	0	0	0	0	0	0	0	0	0	0	0	0	0	1	2	1	1		
	2	RCT	0			0	0				0	0	0	0	0	0	0	0	0	0	0	0	1	0	1	2	1	1		
**McCoy 2017b**	1	Quasi	1	438	1	0	0	Africa	ART Hx	1	0	0	0	0	0	0	0	0	0	0	0	0	0	0	1	1	1	0		
**McLaughlin 2018**	1	RCT	0	356	1	0	0	South America	Mixed	0	0	0	0	0	0	0	0	1	0	1	0	0	0	0	1	3	1	0		
**Mills 2018**	1	RCT	0	2170	1	0	0	Africa	ART Hx	1	1	0	0	0	0	0	0	0	0	0	0	0	0	0	0	1	0	0		
	2	RCT	0			0	0				1	0	0	0	0	0	0	0	0	0	0	0	0	0	1	2	0	0		
**Mimiaga 2019**	1	RCT	1		0	0	0	North America	Unclear	0	0	1	0	0	0	1	0	0	0	0	0	0	0	0	0	2	1	1		

Supplementary material File C. Studies included in hybrid-narrative review[1-231]

STUDY FEATURES											INTERVENTON STRATEGIES																OUTCOMES			
First Author and date	Intervention*	Design	Pilot	N	Powered	SR Only	Has VL	Location(s)	ART Status	General	Economic strategies	eHealth/ mHealth	Alcohol/drug use	Adherence clubs	Adherence education	Adherence counseling	MH counseling	Peer support	Electronic dose monitoring	Directly observed therapy	Medication scheduling	Regimen simplification	Food support	Task shifting	Other	**Total Strategies**	Any evidence of effect	Significant impact	Any evidence of effect: VL	Significant impact: VL
**Molemans 2019**	1	Quasi	0	191	1	1	0	Africa	ART naïve	1	0	0	0	0	0	0	0	0	0	0	0	0	0	0	1	1	0	0		
**Monroe 2018**	1	RCT	1	46	0	0	0	North America	ART Hx	1	0	0	0	0	0	0	0	0	0	0	0	0	0	0	1	1	0	0		
**Moore 2015**	1	RCT	0	58	0	0	0	North America	ART Hx	0	0	1	0	0	0	1	1	0	0	0	0	0	0	0	0	3	1	0		
**Moore 2018**	1	RCT	1	66	0	0	0	North America	ART Hx	0	0	1	0	0	0	1	0	0	0	0	0	0	0	0	1	3	0	0		
**Mutambanengwe-Jacob 2022**	1	RCT	1	50	1	0	1	Africa	ART Hx	0	0	0	0	1	0	0	0	0	0	0	0	0	0	0	1	2	0	0	0	0
**Myer 2018**	1	RCT	0	471	1	0	1	Africa	ART Hx	0	0	0	0	0	0	0	0	0	0	0	0	0	0	0	1	1	1	1	1	1
**Myer 2022**	1	RCT	0	409	1	0	1	Africa	ART Hx	0	0	0	1	0	0	0	0	0	0	0	0	0	0	0	0	1	1	0	1	0
**Naar 2020**	1	RCT	0	183	1	0	1	North America	ART Hx	0	0	0	0	1	0	0	0	0	0	0	0	0	0	0	1	2	0	0	0	0
**Nakimuli-Mpungu 2022**	1	RCT	0	1140	1	0	1	Africa	ART Hx	0	0	0	0	0	0	0	1	0	0	0	0	0	0	1	1	3	1	1	1	1
**Nance 2017**	1	RCT	0		1	0	0	Africa	Unclear	0	0	0	0	0	0	1	1	1	0	0	0	0	0	0	1	4	1	0		
**Nanyeenya 2023**	1	RCT	0	136	1	0	1	Africa	Unclear	1	0	0	0	0	1	0	1	0	0	0	0	0	0	0	0	2	1	1	1	1

Supplementary material File C. Studies included in hybrid-narrative review[1-231]

STUDY FEATURES											INTERVENTON STRATEGIES																OUTCOMES			
First Author and date	Intervention*	Design	Pilot	N	Powered	SR Only	Has VL	Location(s)	ART Status	General	Economic strategies	eHealth/ mHealth	Alcohol/drug use	Adherence clubs	Adherence education	Adherence counseling	MH counseling	Peer support	Electronic dose monitoring	Directly observed therapy	Medication scheduling	Regimen simplification	Food support	Task shifting	Other	**Total Strategies**	Any evidence of effect	Significant impact	Any evidence of effect: VL	Significant impact: VL
**Ndenkeh JJN 2022**	1	RCT	0	370	1	0	1	Africa	ART Hx	1	0	0	0	0	0	0	1	0	0	0	0	0	0	0	0	1	1	0	1	0
**Ndhlovu 2021**	1	RCT	0	214	0	0	1	Africa	Unclear	0	0	1	0	0	0	0	0	1	0	0	0	0	0	0	1	3	1	1	1	1
**Neumann 2018**	1	Quasi	0	4003	1	0	1	North America	Unclear	1	0	0	0	0	0	0	0	0	0	0	0	0	0	0	1	1	1	1	1	1
**Ngcobo 2022**	1	Quasi	0	558	1	0	1	Africa	ART naïve	1	0	0	0	0	1	0	0	0	0	0	0	0	0	0	1	1	0	0	0	0
	2	Quasi	0			0	1				0	0	0	0	1	0	0	1	0	0	0	0	0	0	1	3	0	0	0	0
	3	Quasi	0			0	1				0	0	0	0	1	0	0	1	0	0	0	0	0	0	1	3	0	0	0	0
**Novak 2023**	1	RCT	0	102	0	0	1	North America	Unclear	1	1	0	0	0	0	0	0	0	0	0	0	0	0	0	0	1	1	1	1	1
**Nsagha 2016**	1	RCT	0	90	1	1	0	Africa	ART Hx	1	0	1	0	0	1	0	0	0	0	0	0	0	0	0	0	2	1	1		
**Olashore 2023**	1	RCT	0	50	1	1	0	Africa	ART Hx	0	0	0	0	0	0	1	1	0	0	0	0	0	0	0	1	3	1	0		
**Orrell 2015**	1	RCT	0	230	0	0	0	Africa	ART naïve	0	0	1	0	0	0	0	0	0	1	0	0	0	0	0	1	3	1	0		
**Palar 2017**	1	Pre Post Single Arm	0	72	0	1	0	North America	Unclear	0	0	0	0	0	0	0	0	0	0	0	0	0	1	0	0	1	1	1		

Supplementary material File C. Studies included in hybrid-narrative review[1-231]

STUDY FEATURES											INTERVENTON STRATEGIES																OUTCOMES			
First Author and date	Intervention*	Design	Pilot	N	Powered	SR Only	Has VL	Location(s)	ART Status	General	Economic strategies	eHealth/ mHealth	Alcohol/drug use	Adherence clubs	Adherence education	Adherence counseling	MH counseling	Peer support	Electronic dose monitoring	Directly observed therapy	Medication scheduling	Regimen simplification	Food support	Task shifting	Other	**Total Strategies**	Any evidence of effect	Significant impact	Any evidence of effect: VL	Significant impact: VL
**Pang 2020**	1	RCT	1	40	0	0	0	Asia	ART Hx	1	1	1	0	0	0	0	0	0	0	1	0	0	0	0	1	4	0	0		
**Parsons 2018**	1	RCT	0		0	0	1	North America	ART Hx	0	0	0	0	0	0	1	0	0	0	0	0	0	0	0	0	1	1	0	0	0
**Pascoe 2019**	1	RCT	0	730	1	0	1	Africa	ART naïve	1	0	0	0	0	1	1	0	0	0	0	0	0	0	0	0	3	1	0	1	0
**Peltzer 2017**	1	RCT	0	699	1	0	0	Africa	Unclear	0	0	0	0	0	0	1	0	0	0	0	0	0	0	0	0	1	0	0		
**Pence 2015**	1	RCT	0	304	1	0	0	North America	Unclear	1	0	0	0	0	0	1	0	0	0	0	0	0	0	0	1	2	0	0		
**Pfeiffer 2017**	1	RCT	0	761	1	0	0	Africa	ART naïve	0	0	0	0	0	1	0	0	0	0	0	0	0	0	0	1	2	1	1		
**Pokhrel 2018**	1	Quasi	0	682	1	1	0	Asia	ART Hx	1	0	0	0	0	1	0	0	1	0	0	0	0	0	0	1	3	1	1		
**Psaros 2023**	1	RCT	1	23	0	0	0	Africa	ART naïve	0	0	0	0	0	0	1	1	0	0	0	0	0	0	0	0	2	0	0		
**Ramsey 2021**	1	RCT	1	53	0	0	1	North America	ART Hx	1	0	1	0	0	0	1	1	0	0	0	0	0	0	0	1	4	1	0	1	0
**ReidMJA 2017**	1	RCT	0	108	1	0	1	Africa	ART Hx	1	0	1	0	0	0	0	0	0	0	0	0	0	0	0	1	2	1	0	0	0
**Reif 2022**	1	RCT	0	150	1	0	1	North America	ART Hx	0	0	0	0	0	1	0	0	0	0	0	0	0	0	0	1	2	0	0	0	0

Supplementary material File C. Studies included in hybrid-narrative review[1-231]

STUDY FEATURES											INTERVENTON STRATEGIES																OUTCOMES			
First Author and date	Intervention*	Design	Pilot	N	Powered	SR Only	Has VL	Location(s)	ART Status	General	Economic strategies	eHealth/ mHealth	Alcohol/drug use	Adherence clubs	Adherence education	Adherence counseling	MH counseling	Peer support	Electronic dose monitoring	Directly observed therapy	Medication scheduling	Regimen simplification	Food support	Task shifting	Other	**Total Strategies**	Any evidence of effect	Significant impact	Any evidence of effect: VL	Significant impact: VL
**Robbins 2015**	1	RCT	1	65	0	0	0	Africa	ART Hx	1	0	1	0	0	0	1	1	0	0	0	0	0	0	0	0	3	1	0		
**Ronen 2023**	1	Pre Post Single Arm	1	55	1	1	0	Africa	ART Hx	0	0	1	0	0	0	1	1	1	0	0	0	0	0	0	0	4	1	0		
**Ross-Degnan 2017**	1	RCT	0	3150	1	0	0	Africa	ART Hx	0	0	0	0	0	0	0	0	0	0	0	0	0	0	0	1	1	0	0		
**Rotheram-Borus 2023**	1	RCT	0	297	1	1	0	Africa	Unclear	0	0	0	0	0	0	0	0	0	0	0	0	0	0	0	1	1	1	1		
**Rotzinger 2016**	1	Pre Post Single Arm	0	88	0	0	1	Europe	ART Hx	1	0	0	0	0	0	0	0	0	0	0	0	1	0	0	0	1	0	0	0	0
**Rowell-Cunsolo 2020**	1	RCT	1	32	0	0	1	North America	ART Hx	0	0	0	0	0	1	1	1	1	0	0	0	0	0	0	0	4	1	0	1	0
**Ruan 2017**	1	RCT	0	100	0	0	0	Asia	ART Hx	1	0	1	0	0	1	1	1	0	0	0	0	0	0	0	0	4	1	1		
**Ruel 2023**	1	RCT	0	1549	1	0	1	Africa	Mixed	0	0	1	0	0	0	1	0	0	0	0	0	0	0	0	1	3	1	1	1	1
**Saberi 2021**	1	RCT	1	50	0	0	0	North America	Unclear	0	0	1	0	0	1	0	1	0	0	0	0	0	0	0	0	3	1	0		
**Sabin 2015**	1	RCT	0	116	1	0	1	Asia	Mixed	1	0	1	0	0	0	1	0	0	1	0	0	0	0	0	1	4	1	1	0	0
**Sabin 2022**	1	RCT	0	131	1	0	1	Africa	ART naïve	0	0	1	0	0	0	1	0	0	1	0	0	0	0	0	0	3	1	0	1	0

Supplementary material File C. Studies included in hybrid-narrative review[1-231]

STUDY FEATURES											INTERVENTON STRATEGIES																OUTCOMES			
First Author and date	Intervention*	Design	Pilot	N	Powered	SR Only	Has VL	Location(s)	ART Status	General	Economic strategies	eHealth/ mHealth	Alcohol/drug use	Adherence clubs	Adherence education	Adherence counseling	MH counseling	Peer support	Electronic dose monitoring	Directly observed therapy	Medication scheduling	Regimen simplification	Food support	Task shifting	Other	**Total Strategies**	Any evidence of effect	Significant impact	Any evidence of effect: VL	Significant impact: VL
**Safren 2016**	1	RCT	0	240	1	0	1	North America	Unclear	0	0	0	0	0	1	1	1	1	0	0	0	0	0	0	0	4	0	0	0	0
**Safren 2021**	1	RCT	0	160	1	0	1	Africa	Unclear	0	0	0	0	0	0	0	1	0	0	0	0	0	0	1	0	2	1	1	1	1
**Sakthivel 2022**	1	RCT	0	388	1	0	0	Asia	ART Hx	0	0	0	0	0	0	0	0	0	0	0	0	0	0	0	1	1	1	1		
**Sanchez 2021**	1	RCT	0	143	0	1	0	Guatamala	Unclear	0	0	1	0	0	0	0	0	0	0	0	0	0	0	0	1	2	1	0		
**Sanchez-Dominguez 2020**	1	Quasi	0	3661	1	1	0	Central America	Unclear	1	0	0	0	0	1	0	0	0	0	0	0	0	0	0	1	2	0	0		
**Sarna 2019**	1	RCT	0	404	1	0	0	Africa	Mixed	0	0	0	0	0	0	1	0	0	0	0	0	0	0	0	0	1	0	0		
**Satre 2019**	1	RCT	0	614	1	0	1	North America	Unclear	0	0	1	1	0	1	1	0	0	0	0	0	0	0	0	0	4	0	0	0	0
	2	RCT	0			0	1				0	1	1	0	0	0	0	0	0	0	0	0	0	0	0	4	0	0	0	0
**Scharer 2020**	1	RCT	1	34	0	0	1	North America	ART naïve	0	0	0	0	0	0	1	1	0	0	0	0	0	0	0	0	2	0	0	0	0
**Schensul 2021**	1	RCT	0	871	1	0	1	Asia	ART Hx	0	0	1	0	0	0	1	1	0	0	0	0	0	0	0	1	2	1	1	1	1
	2	RCT	0			0	1				0	0	0	0	1	1	0	1	0	0	0	0	0	0	0	3	1	1	0	0

Supplementary material File C. Studies included in hybrid-narrative review[1-231]

STUDY FEATURES											INTERVENTON STRATEGIES																OUTCOMES			
First Author and date	Intervention*	Design	Pilot	N	Powered	SR Only	Has VL	Location(s)	ART Status	General	Economic strategies	eHealth/ mHealth	Alcohol/drug use	Adherence clubs	Adherence education	Adherence counseling	MH counseling	Peer support	Electronic dose monitoring	Directly observed therapy	Medication scheduling	Regimen simplification	Food support	Task shifting	Other	**Total Strategies**	Any evidence of effect	Significant impact	Any evidence of effect: VL	Significant impact: VL
	3	RCT	0			0	1				0	0	0	0	0	0	0	1	0	0	0	0	0	0	1	2	0	0	0	0
**Schnall 2018**	1	RCT	0	80	0	1	0	North America	Unclear	1	0	1	0	0	1	0	0	0	0	0	0	0	0	0	1	3	1	1		
**Schnall 2023**	1	RCT	0	198	1	0	1	North America	Unclear	1	0	1	0	0	1	0	0	0	1	0	0	0	0	0	1	0	1	0	0	0
**Sevelius 2022**	1	RCT	0	278	0	0	0	North America	Unclear	0	0	0	0	0	0	0	0	1	0	0	0	0	0	0	1	2	1	1		
**Sherman 2020**	1	RCT	1	94	0	0	1	North America	ART naïve	1	0	1	0	0	0	0	0	0	0	0	0	0	0	0	1	2	1	0	1	0
**Shim 2022**	1	RCT	1	33	1	1	0	Asia	ART Hx	1	0	1	0	0	1	1	0	1	0	0	0	0	0	0	1	5	1	0		
**Sibinga 2022**	1	RCT	0	74	0	0	1	North America	Unclear	0	0	0	0	0	0	0	0	0	0	0	0	0	0	0	1	1	0	0	0	0
**Sikkema 2018**	1	RCT	1	64	0	0	1	Africa	ART naïve	0	0	0	0	0	0	0	1	0	0	0	0	0	0	0	0	1	0	0	0	0
**Silverman 2019**	1	RCT	0	102	1	0	1	North America	Mixed	1	1	0	0	0	0	0	0	0	0	0	0	0	0	0	0	1	1	1	1	1
**Spelke 2022**	1	RCT	1	80	0	0	1	Africa	ART Hx	0	0	0	0	0	0	0	1	0	0	0	0	0	0	0	0	1	0	0	0	0
**Spratt 2017**	1	RCT	1	6	0	0	1	North America	Unclear	0	0	1	0	0	1	1	0	0	0	0	0	0	0	0	1	4	0	0	0	0

Supplementary material File C. Studies included in hybrid-narrative review[1-231]

STUDY FEATURES											INTERVENTON STRATEGIES																OUTCOMES			
First Author and date	Intervention*	Design	Pilot	N	Powered	SR Only	Has VL	Location(s)	ART Status	General	Economic strategies	eHealth/ mHealth	Alcohol/drug use	Adherence clubs	Adherence education	Adherence counseling	MH counseling	Peer support	Electronic dose monitoring	Directly observed therapy	Medication scheduling	Regimen simplification	Food support	Task shifting	Other	**Total Strategies**	Any evidence of effect	Significant impact	Any evidence of effect: VL	Significant impact: VL
	2	RCT	1			0	1				0	1	0	0	0	1	0	0	1	0	0	0	0	0	0	4	1	0	0	0
**Ssewamala 2020**	1	RCT	0	288	0	0	1	Africa	ART Hx	0	1	0	0	0	1	0	0	0	0	0	0	0	0	0	0	2	1	1	1	1
**Stephenson 2021**	1	RCT	0	318	1	1	0	North America	ART Hx	0	0	0	0	0	1	1	1	0	0	0	0	0	0	0	1	4	1	1		
**Sued 2022**	1	RCT	0	360	1	0	1	South America	ART Hx	1	0	0	0	0	0	1	0	0	0	0	0	0	0	0	0	1	1	0	1	0
**Sumari-deBoer 2021**	1	RCT	0	249	1	0	1	Africa	Unclear	1	0	1	0	0	0	1	0	0	1	0	0	0	0	0	1	4	0	0	0	0
	2	RCT	0			0	1				0	1	0	0	0	0	0	0	0	0	0	0	0	0	1	2	0	0	0	0
**Swendeman 2015**	1	Pre Post Single Arm	1	46	0	1	0	Asia	ART Hx	1	0	1	0	0	1	0	0	0	0	0	0	0	0	0	1	3	1	1		
**Swendeman 2020**	1	RCT	1	362	1	0	0	Asia	ART Hx	1	0	1	0	0	1	0	0	0	0	0	0	0	0	0	0	2	0	0		
**Thirumurthy 2019**	1	RCT	0	400	1	0	1	Africa	Mixed	1	1	0	0	0	0	0	0	0	0	0	0	0	0	0	0	1	1	0	1	0
**Tozan 2021**	1	RCT	0	702	1	0	1	Africa	ART Hx	0	1	0	0	0	0	0	0	0	0	0	0	0	0	0	0	1	1	1	1	1
**Tran 2023**	1	RCT	0	495	1	1	0	Africa; Asia	ART Hx	1	0	1	1	0	1	0	0	0	0	0	0	0	0	0	1	4	1	1		

Supplementary material File C. Studies included in hybrid-narrative review[1-231]

STUDY FEATURES											INTERVENTON STRATEGIES																OUTCOMES			
First Author and date	Intervention*	Design	Pilot	N	Powered	SR Only	Has VL	Location(s)	ART Status	General	Economic strategies	eHealth/ mHealth	Alcohol/drug use	Adherence clubs	Adherence education	Adherence counseling	MH counseling	Peer support	Electronic dose monitoring	Directly observed therapy	Medication scheduling	Regimen simplification	Food support	Task shifting	Other	**Total Strategies**	Any evidence of effect	Significant impact	Any evidence of effect: VL	Significant impact: VL
**Tukei 2020**	1	RCT	0	5336	1	0	1	Africa	ART Hx	1	0	0	0	0	0	0	0	0	0	0	1	0	0	0	1	2	1	1	1	1
	2	RCT	0			0	1				0	0	0	1	0	0	0	0	0	0	1	0	0	0	1	2	1	1	1	1
**Tull 2018**	1	Pre Post Single Arm	1	10	0	1	0	North America	ART Hx	1	0	0	0	0	0	1	1	0	0	0	0	0	0	0	0	2	0	0		
**Uusküla 2018**	1	RCT	0	519	1	0	1	Europe	Mixed	1	0	0	0	0	0	1	0	0	0	0	0	0	0	0	0	1	1	1	0	0
**Vance 2023**	1	RCT	0	216	1	1	0	North America	ART Hx	1	0	0	0	0	0	0	0	0	0	0	0	0	0	0	1	1	1	1		
	2	RCT	0			1	0				0	0	0	0	0	0	0	0	0	0	0	0	0	0	1	1	1	1		
**vanElsland 2018**	1	RCT	0	195	0	0	0	Africa	ART Hx	0	0	0	0	0	1	0	0	0	0	0	0	0	0	0	0	1	0	0		
**vanLoggerenberg 2015**	1	RCT	0	297	1	0	1	Africa	ART naïve	1	0	0	0	0	1	1	0	0	0	0	0	0	0	0	0	2	0	0	0	0
**Velvanathan 2016**	1	RCT	0	120	0	0	1	Asia	ART Hx	1	0	0	0	0	0	0	0	0	0	0	0	1	0	0	0	1	1	1	0	0
**Wagner 2021**	1	RCT	0	176	0	0	1	North America	ART naïve	1	0	0	0	0	1	1	0	1	0	0	0	0	0	0	0	3	1	1	1	0
**Wagner 2023**	1	RCT	0	391	1	0	1	Africa	ART Hx	0	0	0	0	0	0	1	0	1	0	0	0	0	0	0	1	3	0	0	0	0

Supplementary material File C. Studies included in hybrid-narrative review[1-231]

STUDY FEATURES											INTERVENTON STRATEGIES															OUTCOMES			
First Author and date	Intervention*	Design	Pilot	N	Powered	SR Only	Has VL	Location(s)	ART Status	General	Economic strategies	eHealth/ mHealth	Alcohol/drug use	Adherence clubs	Adherence education	Adherence counseling	MH counseling	Peer support	Electronic dose monitoring	Directly observed therapy	Medication scheduling	Regimen simplification	Food support	Task shifting	Other	**Total Strategies**	Any evidence of effect	Significant impact	Any evidence of effect: VL	Significant impact: VL
**Webb 2018**	1	RCT	1	72	0	0	1	North America	ART Hx	0	0	0	0	0	0	0	0	0	0	0	0	0	0	0	1	1	1	1	1	1
**Wechsberg 2019**	1	RCT	0	290	0	0	1	Africa	Mixed	0	0	0	1	0	0	0	1	0	0	0	0	0	0	0	0	2	1	1	1	1
**Weiser 2015**	1	RCT	1	140	0	0	1	Africa	ART Hx	1	1	0	0	0	0	0	0	0	0	0	0	0	0	0	0	1	1	1	1	1
**Weissman 2016**	1	RCT	0	142	0	0	1	North America	ART Hx	1	0	0	0	0	0	0	0	0	0	0	1	0	0	0	0	1	0	0	0	0
**White 2015**	1	RCT	1	43	0	0	1	North America	Mixed	0	0	0	0	0	0	0	0	0	0	1	0	0	0	0	0	1	0	0	0	0
**Whiteley 2018**	1	RCT	0	61	0	0	1	North America	Mixed	0	0	1	0	0	1	0	0	0	1	0	0	0	0	0	1	4	1	1	1	0
**Willis 2019**	1	RCT	0	94	0	1	0	Africa	ART Hx	0	0	0	0	0	1	0	1	1	0	0	0	0	0	0	0	3	1	1		
**Wimberly 2020**	1	RCT	0	75	0	0	1	North America	ART Hx	0	0	0	0	0	0	0	0	0	0	0	0	0	0	0	1	1	0	0	0	0
**Wohl 2017**	1	RCT	0	405	1	0	1	North America	Unclear	0	1	0	0	0	1	1	0	0	0	0	0	0	0	0	0	3	1	0	1	0
**Yator 2022**	1	RCT	1	24	0	1	0	Africa	ART Hx	0	0	0	0	0	0	0	1	1	0	0	0	0	0	0	0	2	0	0		
**Yotebieng 2016**	1	RCT	0	433	0	0	1	Africa	ART naïve	0	1	0	0	0	0	0	0	0	0	0	0	0	0	0	0	1	0	0	0	0

TABLE KEY

Term	Meaning
Intervention*	Intervention(s) characterized in the study<br>1= A single intervention evaluated<br>2= 2nd intervention evaluated<br>3= 3rd intervention evaluated<br>*Overall study information is presented in line '1'
Design	Type of intervention study
Pilot	1= Was presented as a pilot<br>0= Was NOT presented as a pilot
N	Sample size (blank=could not be determined)
Powered	1= Reported being powered for primary outcome or judged to be sufficiently powered (e.g., 300 or more participants) if not specifically reported<br>0= Reported insufficient power, power not appropriate given pilot aims, or not stated and sample size was less than 300
SR Only	1= Only used self-reported adherence for outcome<br>0= Any other combination of outcomes
Has VL	1= Included viral load-based outcome(s)<br>0= Did not report on viral load-based outcome
Location(s)	Continent/Location of participants
ART Status	Inclusion Criteria around experience with ART<br>Naïve = New to ART<br>ART HX= Had to have a history with ART (not new)<br>Mixed= Sample included both<br>Unclear= Could not determine
General	1= Study was conducted with a general clinic sample/general sample (e.g., adherence problem not an inclusion criteria)<br>0= Other sample approach
Economic strategies	Study Arm included an economic focused intervention (incentives, micro-finance)
eHealth/ mHealth	Study Arm included an eHealth component (SMS, cell phone, webpage, app)
Alcohol/drug use	Study Arm included intervention strategies focused on addressing alcohol or drug use
Adherence clubs	Study Arm included use of an adherence club
Adherence education	Study Arm included adherence education (training, class, information)
Adherence counseling	Study Arm included adherence counseling (counseling approaches adapted to adherence to ART)
MH counseling	Study Arm included Mental Health support or counseling (can include addressing adherence but primary focus is on improvement of mental health and well-being)
Peer support	Study Arm included peers (e.g., for delivery of intervention, linking peers together, peer groups)
Electronic dose monitoring	Study Arm used Electronic Dose Monitoring as an intervention
Directly observed therapy	Study Arm included Directly observed therapy (in person, adherence supporter or virtual)

Medication scheduling	Study Arm included working to improve regimen dosing schedule, dispensation schedule
Regimen simplification	Study Arm included regimen simplification
Food support	Study Arm included strategies to mitigate food insecurity
Task shifting	Study Arm included shifting provision of care/services to cadres that may be better resourced to provide service
Other	Study Arm included other unique intervention strategies
Total Strategies	Total number of strategies coded for the intervention (arm)
Any Support	1= Intervention (arm) was supported in some way (promising or in direction anticipated in pilots, trends for powered studies) on any adherence related outcome<br>0= Intervention (arm) not supported
Sig Effect Any	1= Intervention (arm) was reported significant impact on one or more outcomes<br>0= Intervention (arm) did not have significant impact on any of the adherence-related outcomes
Any Support VL	1= Intervention (arm) was supported (promising or in direction anticipated in pilots, trends for powered studies) on any viral load outcome<br>0= Intervention (arm) not supported on any viral load outcome<br>*Characterized only for studies that included a viral load outcome: Blank=Did not include
Sig Effect VL	1= Intervention (arm) had significant impact on one or more viral load outcome<br>0= Intervention (arm) did not have significant impact on any viral load impact<br>*Characterized only for studies that included a viral load outcome: Blank=Did not include
